# ER‐phagy Activation by AMFR Attenuates Cardiac Fibrosis Post‐Myocardial Infarction via mTORC1 Pathway

**DOI:** 10.1002/advs.202504552

**Published:** 2025-07-17

**Authors:** Zhixiang Wang, Kaifan Niu, Wei Liu, Xinyun Wang, Boshen Yang, Taixi Li, Yizhi Chen, Yuanyuan Jin, Yu Chen, Yangyi Lin, Xian Jin

**Affiliations:** ^1^ Department of Cardiology Shanghai Sixth People's Hospital Affiliated to Shanghai Jiao Tong University School of Medicine Shanghai 200233 China; ^2^ Tongren Hospital, Shanghai Jiao Tong University School of Medicine Shanghai 200050 China; ^3^ International Medical College of Chongqing Medical University Chongqing 400010 China

**Keywords:** AMFR, ER‐phagy, FAM134B, fibroblast activation, mTORC1, myocardial infarction

## Abstract

Progressive cardiac fibrosis post myocardial infarction (MI) drives pathological remodeling and heart failure, yet the role of endoplasmic reticulum‐selective autophagy (ER‐phagy) in this process remains unclear. Autocrine Motility Factor Receptor (AMFR) is a recently identified ER‐phagy regulator, whose function under myocardial pathology remains poorly understood. Here, it is found that FAM134B‐mediated ER‐phagy activity is elevated in fibrotic mouse heart tissues post‐MI and in cardiac fibroblasts stimulated by TGF‐β1. AMFR knockout in mice aggravated cardiac fibrosis post‐MI and worsened cardiac function, with scRNA‐seq analysis demonstrating that AMFR‐null cardiac fibroblasts exhibit a myofibroblast phenotype. Simultaneously, AMFR overexpression in cardiac fibroblasts reduces the expression of profibrogenic proteins in response to TGF‐β1 stimulation. AMFR regulates ER‐phagy flux and turnover of FAM134B, which leads to the suppression of cardiac fibroblasts activation. Mechanistically, AMFR catalyzed K27‐linked (predominant) and K33‐linked ubiquitination of FAM134B and enhanced ER‐phagy flux, thereby inhibiting the phosphorylation of mTORC1 downstream targets such as S6K1 and 4E‐BP. These findings highlight the therapeutic potential of AMFR‐driven ER‐phagy in suppressing cardiac fibrosis post‐MI.

## Graphical abstract

By catalyzing FAM134B ubiquitination and activating ER‐phagy, AMFR alleviates progressive fibrosis and cardiac dysfunction by inhibiting the mTORC1 pathway. Consequently, these findings underscore the essential role of AMFR‐driven ER‐phagy in mitigating the progression of fibrotic responses, offering a potential therapeutic target for preventing heart failure post‐MI.

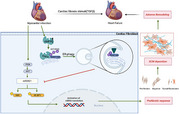

## 1 Introduction

Myocardial infarction (MI) is the most common and critical disease among cardiovascular and cerebrovascular diseases.^[^ ^1^ ^]^ Although the myocardial reperfusion therapy has significantly reduced the mortality of MI, a large proportion of survivors will develop heart failure due to adverse ventricular remodeling.^[^ ^2^ ^]^ Pathological fibrosis, characterized by excessive deposition of extracellular matrix (ECM), is generally recognized as central to ventricular remodeling post‐MI.^[^ ^3^ ^]^ Notably, activated fibroblasts, the main producers of matrix proteins, are the primary cellular effectors in cardiac fibrosis. Thus, elucidating the mechanisms of cardiac fibroblasts underlying cardiac remodeling post‐MI is crucial for developing targeted therapeutic interventions.

The endoplasmic reticulum (ER) is essential for protein synthesis and modification in eukaryotic cells.^[^ ^4^, ^5^ ^]^ Current studies suggest that prolonged ER stress may lead to fibrosis through activation of CCAAT/enhancer‐binding homologous protein‐mediated apoptosis, followed by an inflammatory response and release of profibrotic cytokines.^[^ ^6^ ^]^ Endoplasmic reticulum selectively autophagy (ER‐phagy), a quality control mechanism that degrades damaged ER subdomains through the lysosomal pathway, alleviates ER stress and restores ER homeostasis.^[^ ^7^ ^]^ This selective clearance helps not only to eliminate abnormal ER and proteins, but also to prevent further cellular damage and provide cells with necessary nutrients and energy,^[^ ^8^, ^9^ ^]^ which may affect activation of fibroblasts. Recently, ER‐phagy receptors in mammals have been discovered successively, including FAM134B, ATL3, SEC62, CCPG1, TEX264, CALCOCO1, etc.^[^ ^10^, ^11^, ^12^, ^13^, ^14^, ^15^ ^]^ It has been reported that ER‐phagy is favorable to diseases such as lung injury and kidney injury.^[^ ^16^, ^17^ ^]^ ER‐phagy has also been found to alleviate anthracycline‐induced cardiotoxicity through CCPG1‐mediated signaling.^[^ ^18^ ^]^ However, the role of ER‐phagy in the process of ventricular remodeling post‐MI and the mechanisms that regulate ER‐phagy have not been investigated.

Autocrine motility factor receptor (AMFR), an E3 ubiquitin ligase primarily expressed on the ER membrane,^[^ ^19^ ^]^ is recently been identified as an ER‐regulator that catalyzes FAM134B ubiquitination.^[^ ^20^ ^]^ It has been reported that AMFR regulates protein degradation and serves critical roles in biological processes such as inflammatory responses,^[^ ^21^, ^22^ ^]^ innate immunity,^[^ ^23^ ^]^ allergic asthma,^[^ ^24^ ^]^ and so on. However, existing research has not yet involved the regulatory role of AMFR in cardiovascular diseases.

Therefore, this study aims to determine the activation status of ER‐phagy post‐MI and its association with fibrotic progression, investigate the role of AMFR in coordinating ER‐phagy and fibroblast activation, and elucidate the molecular mechanisms underlying AMFR‐mediated modulation of these processes. We found that ER‐phagy was activated in cardiac fibroblasts post‐myocardial infarction. AMFR alleviated cardiac fibrosis post‐MI through activation of ER‐phagy. Mechanistically, AMFR ubiquitinated FAM134B to activate ER‐phagy, thereby inhibiting the mTORC1 signaling pathway in cardiac fibroblasts. Our study identifies AMFR as an ER‐phagy regulator that suppresses cardiac fibrosis by promoting ubiquitination of FAM134B, and provides the first evidence of the crucial role of AMFR in inhibiting the mTORC1 pathway.

## 2 Results

### 2.1 FAM134B‐Mediated ER‐phagy is Activated in Cardiac Fibroblasts Post‐MI

**Figure 1:**
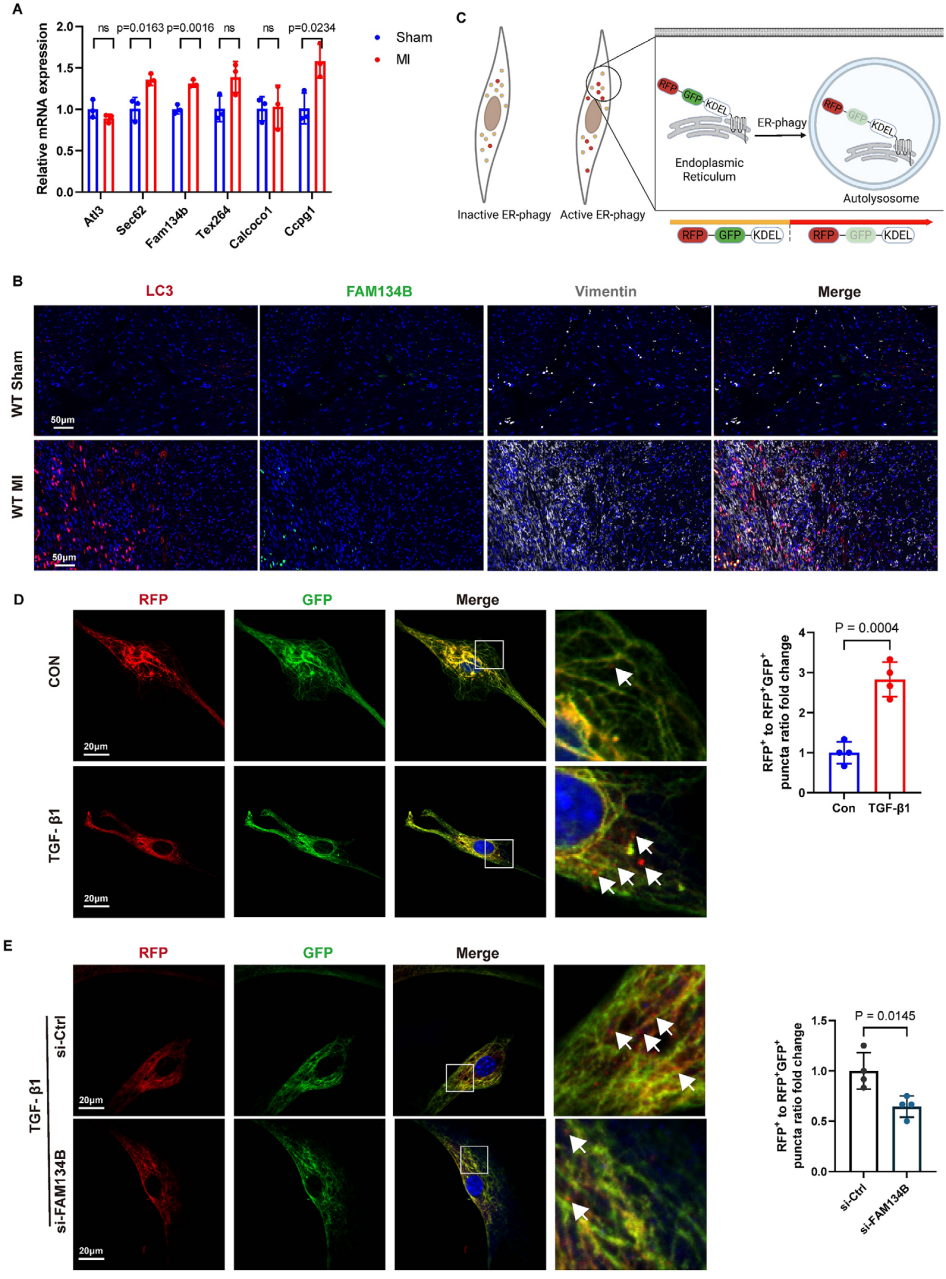
FAM134B‐mediated ER‐phagy is activated in cardiac fibroblasts post‐MI. A) The relative mRNA levels of Atl3, Sec62, Fam134b, Tex264, Calcoco1, and Ccpg1 in the mouse heart tissue sections from sham or MI mice at day 7 post‐MI. n = 3. B) Immunofluorescence co‐staining of LC3 (red) with FAM134B (green), Vimentin (white), and DAPI (blue) in the mouse heart tissue sections from sham or MI mice at day 7 post‐MI. C) Regimen of RFP‐GFP‐KDEL. D) Fluorescent imaging of RFP–GFP–KDEL transfected adult mouse primary cardiac fibroblasts (ACFs) isolated from WT mice treated with PBS or TGF‐β1(10 ng mL^−1^) for 24 h. n = 4. E) Fluorescent imaging of RFP–GFP–KDEL transfected ACFs isolated from WT mice treated with si‐Ctrl or si‐FAM134B before treating with TGF‐β1(10 ng mL^−1^) for 24 h. n = 4. Data are presented as mean ± SD. The data shown in A, D, and E were analyzed using an unpaired *t*‐test. MI, myocardial infarction; TGF‐β1, transforming growth factor‐β1; ACFs, adult mouse primary cardiac fibroblasts; WT, wild‐type.

To identify ER‐phagy activation post‐MI, we examined several established ER‐phagy receptors, such as ATL3,^[^ ^14^ ^]^ SEC62,^[^ ^11^ ^]^ FAM134B,^[^ ^10^ ^]^ TEX264,^[^ ^13^ ^]^ CALCOCO1,^[^ ^15^ ^]^ and CCPG1,^[^ ^12^ ^]^ as their increased transcription may indicate enhanced ER‐phagy activity.^[^ ^18^ ^]^ Among these, Fam134b, Ccpg1, and Sec62 were significantly up‐regulated at day 7 post‐MI, with Fam134b being the most prominent (**Figure** **1**A). Increased co‐localization of FAM134B and LC3 was observed in cardiac fibroblasts within the infarct border zone, confirming the activation of ER‐phagy in fibrotic tissues after MI (Figure 1B).

In vitro, ER‐phagy flux was assessed using a dual‐fluorescence reporter, RFP‐GFP‐KDEL, as the RFP/RFP‐GFP puncta ratio reflects ER‐phagy flux due to quenching of GFP in autolysosomes (Figure 1C). Adult mouse primary cardiac fibroblasts (ACFs) isolated from wild‐type (WT) mice were treated with TGF‐β1(10 ng mL^−1^) to simulate the activation of cardiac fibroblasts post‐MI. The results showed increased RFP puncta in ACFs treated with TGF‐β1, indicating enhanced ER‐phagy activity in cardiac fibroblasts after TGF‐β1 stimulation (Figure 1D).

To further determine the role of FAM134B in ER‐phagy, ACFs were transfected with RFP‐GFP‐KDEL or mcherry‐GFP‐FAM134B, respectively, reflecting the total ER‐phagy flux and dedicated FAM134B‐mediated ER‐phagy flux. Then, cells were treated with TGF‐β1(10 ng mL^−1^), and we found that the mCherry/mCherry‐GFP puncta ratio was close to the RFP/RFP‐GFP puncta ratio, indicating FAM134B being the predominant ER‐phagy receptor following TGF‐β1 treatment (Figure S1, Supporting Information). Furthermore, the knockdown of FAM134B by si‐FAM134B abolished the increase of RFP signals induced by TGF‐β1, suggesting that FAM134B could be crucial in regulating TGF‐β1‐induced ER‐phagy in cardiac fibroblasts (Figure 1E). Altogether, these data elucidate that FAM134B‐mediated ER‐phagy is activated in fibrotic mouse heart tissues post‐MI and in cardiac fibroblasts stimulated by TGF‐β1.

### 2.2 AMFR is Up‐Regulated in Fibrotic Mice Heart Tissues After Myocardial Infarction and in Cardiac Fibroblasts Stimulated by TGF‐β1

**Figure 2:**
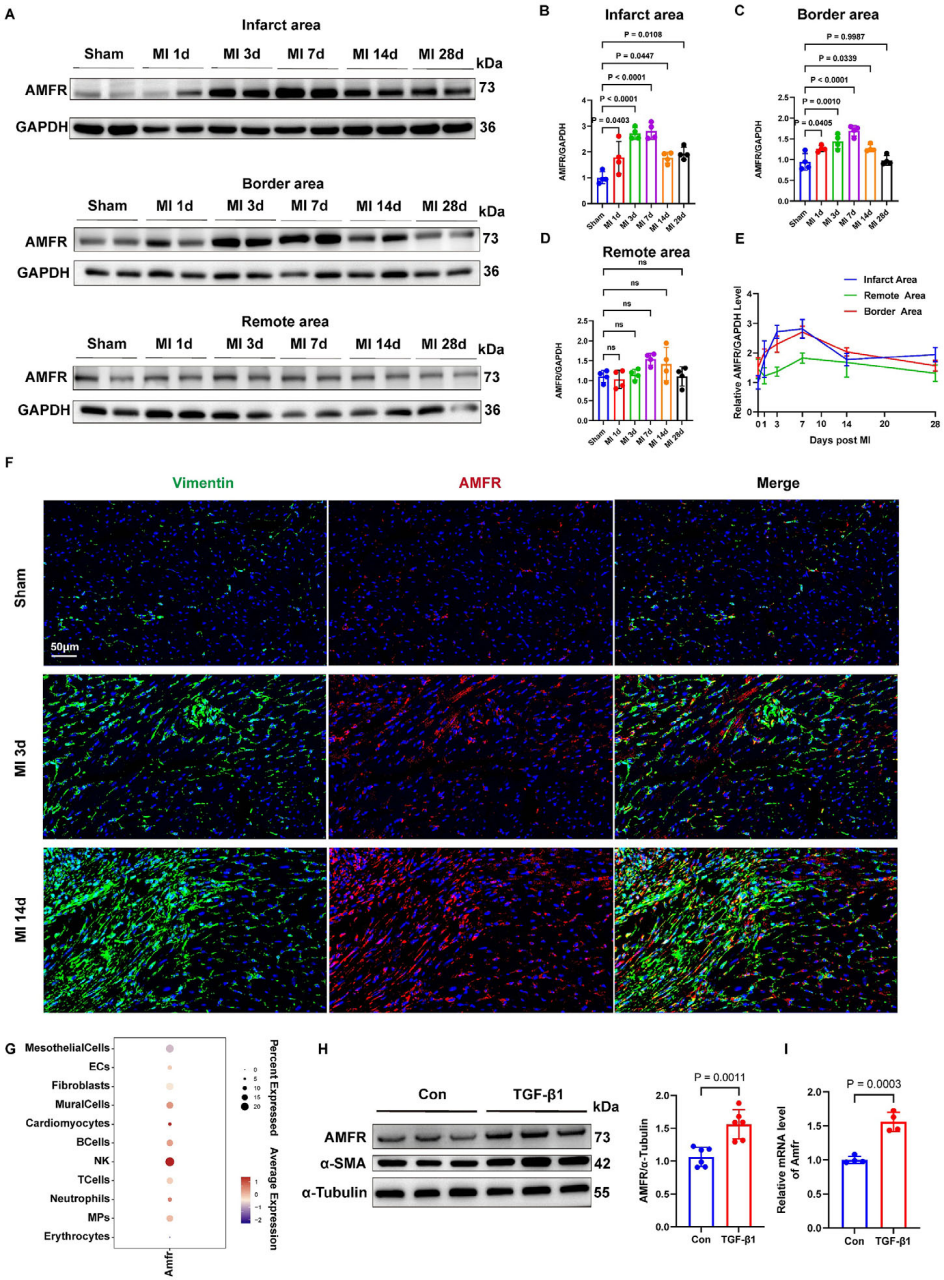
AMFR is up‐regulated in fibroblasts in fibrotic heart tissues. A–D) Expression of AMFR was measured using Western blot analysis in the infarct area, border area, and remote area of WT mice at day 1, day 3, day 7, day 14, and day 28 post‐MI, as well as in sham control. n = 4. E) The XY line graph shows the differences in AMFR expression in the infarct area, border area, and remote area at different time points post myocardial infarction. n = 4. F) Immunofluorescence co‐staining of AMFR (red) with Vimentin (green) and DAPI (blue) in the mouse heart tissue sections from sham mice or MI mice at 3 days, 14 days post MI. G) Amfr expression in different cell types of WT mice at day 7 post‐MI. H) Representative Western blots and quantitative analyses of AMFR and α‐SMA in NRCFs treated with TGF‐β1 (10 ng mL^−1^). n = 6. I) Expression of AMFR was measured using RT‐qPCR in NRCFs treated with TGF‐β1 (10 ng mL^−1^). n = 4. Data are presented as mean ± SD. The data shown in B–D was analyzed using one‐way ANOVA corrected by the post hoc Turkey's test. The data shown in E was analyzed using two‐way ANOVA corrected by the post hoc Turkey's test. The data shown in H and I were analyzed using an unpaired *t*‐test. MI, myocardial infarction; TGF‐β1, transforming growth factor‐β1; NRCFs, neonatal rat cardiac fibroblasts; WT, wild‐type.

Considering the strong association between AMFR and ER‐phagy,^[^ ^20^ ^]^ we investigated the expression level of AMFR at different stages post‐MI. Mice were subjected to permanent left anterior descending artery (LAD) ligation or sham operation and were analyzed at 1 day, 3 days, 7 days, 14 days, and 28 days. At the infarct and border area of heat, the protein levels of AMFR increased from day 3, peaked at day 7 post‐MI, and subsequently decreased but remained higher than those in the sham group at 14 days post‐MI (**Figure** **2**A–D). Concurrently, we compared the protein level of AMFR in the myocardial infarct area, border area, and remote area of tissues. The results showed that there were no significant differences in the expression levels of AMFR in the infarct zone and border zone tissues at all time points. The expression of AMFR in the infarct area was significantly higher than that in the remote area at day 3 post‐MI (p< 0.0001) and day 7 post‐MI (p = 0.0007). The expression of AMFR in the border area was significantly higher than that in the remote area at day 1 post‐MI (p = 0.0009), day 3 post‐MI (p = 0.0001), and day 7 post‐MI (p = 0.0002) (Figure 2E). Consistently, RT‐qPCR indicated that the mRNA level of AMFR increased in the infarcted area 7 days post‐MI, compared to the sham group. The mRNA level of AMFR in the infarcted area and border area was higher than that in the remote zone (Figure S2, Supporting Information). To determine the cell types expressing AMFR during cardiac remodeling, we co‐stained AMFR with Vimentin, a marker of fibroblasts, and observed strong AMFR expression in fibroblasts in the infarct myocardium (Figure 2F). However, we observed less co‐localization of AMFR with the cardiomyocyte marker cTnT, while even less co‐localization of AMFR with the endothelial cell marker CD31 or the macrophage marker CD68 (Figure S2, Supporting Information). Single‐cell RNA‐seq (scRNA‐seq) provided a comprehensive view of AMFR expression across different cardiac cell types, and further confirmed the ubiquitous expression and high abundance of AMFR in cardiac fibroblasts (Figure 2G). Furthermore, we stimulated neonatal rat cardiac fibroblasts (NRCFs) with TGF‐β1, and Western blot analysis and RT‐qPCR showed a significant induction of AMFR expression (Figure 2H,I), indicating AMFR is a novel target responsive to TGF‐β1 in NRCFs. These data demonstrate that AMFR expression is upregulated in activated fibroblasts induced by myocardial infarction and is responsive to TGF‐β1 stimulus.

### 2.3 scRNA‐seq Reveals that AMFR Deletion Promotes Fibroblast Activation and Impairs Autophagy Activity

**Figure 3:**
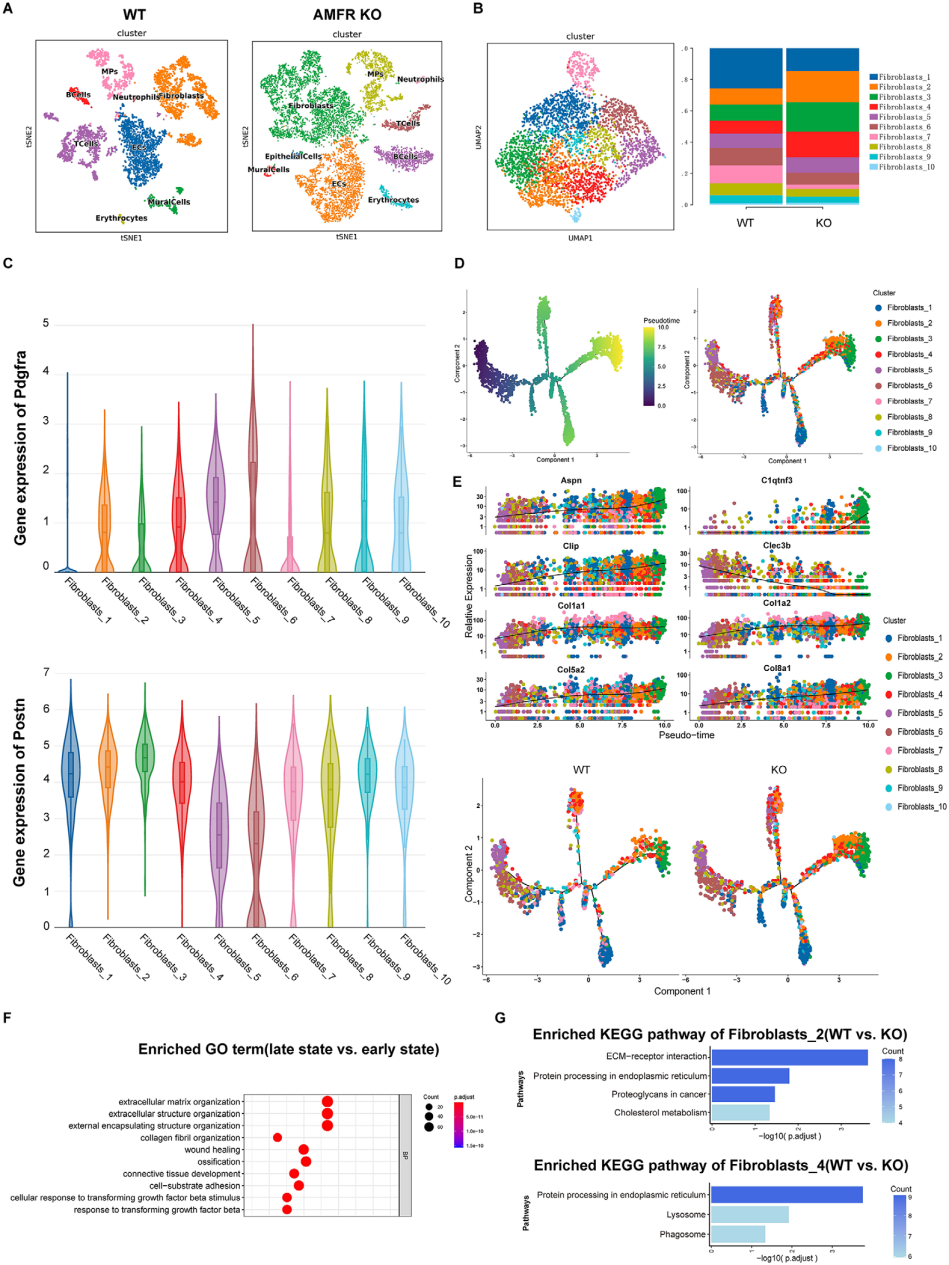
Single cell RNA‐seq reveals that AMFR deletion promotes cardiac fibroblasts activation and impairs autophagy activity. 8‐week‐old WT and AMFR^−/‐^ male mice were subjected to MI surgery, and heart tissues were harvested 1 week post‐surgery for single cell RNA‐seq. A) tSNE of single cell transcriptomes from wild‐type (WT; 5218 cells) and AMFR knockout (KO; 10622 cells) mice obtained 7 days post‐MI. B) UMAP of single cell transcriptomes from WT and AMFR KO fibroblasts, and proportion of each cluster. C) The expression levels of Pdgfra and Postn in 10 clusters of fibroblasts. D) The distribution of 10 clusters of fibroblasts across pseudotime. E) The gene expression of 11 cell states across pseudotime and in different genotypes. F) GO enrichment analysis in late state(state6) and early state(state1). G) KEGG enrichment analysis in WT and KO Fibobalasts_2, as well as WT and KO Fibobalasts_4. MI, myocardial infarction; tSNE, T‐distributed stochastic neighbor embedding; UMAP, Uniform Manifold Approximation and Projection; GO, Gene ontology; KEGG, Kyoto Encyclopedia of Genes and Genomes.

To explore the mechanism of AMFR in response to acute MI, we conducted single‐cell RNA sequencing (scRNA‐seq) on heart tissues isolated from AMFR knockout and WT mice at 7 days post‐MI. After the exclusion of cell doublets based on unique molecular identifier counts and filtering based on mitochondrial genes (Table S1, Supporting Information), we obtained high‐quality sequencing from 5218 WT and 10622 AMFR knockout cells. Nine main cell types were separated following principal component analysis (PCA) and the Louvain clustering algorithm (**Figure** **3**A). Fibroblasts were further subdivided into 10 transcriptionally distinct clusters, with Clusters 2, 3, and 4 significantly increased in the KO group (Figure 3B). By analyzing the expression of key fibrotic marker genes, Cluster 6 was identified as quiescent resident fibroblasts with prominent Pdgfra expression, while Cluster 2, 3 and 4 were identified as activated fibroblasts exhibiting high Postn expression (Figure 3C), with these identities further validated by hierarchical clustering and identification of the most enriched genes in each cluster (Figure S3, Supporting Information).

Pseudotemporal trajectory analysis was then performed to reveal a differentiation continuum from quiescent to activated states (Figure 3D; Figure S3, Supporting Information). Integrating cell identities with the pseudotime trajectory, we observed that Cluster 6 with high Clec3b expression was located at the beginning of pseudotime, while the end of pseudotime was predominantly occupied by Clusters 2, 3, 4, characterized by elevated expression of Aspn, Cilp, Col1a1, Col1a2, Col5a2, Col8a1(Figure 3E). CLEC3B was found to be a potential target for alleviating adipose tissue fibrosis.^[^ ^25^ ^]^ Asporin is an extracellular matrix protein that regulates cardiac remodeling.^[^ ^26^ ^]^ CILP is a Novel Biomarker for Cardiac Fibrosis.^[^ ^27^ ^]^ Col1a1, Col1a2, Col5a2, and Col8a1 encode different types of collagen chains, which play important structural and functional roles in the extracellular matrix.^[^ ^28^, ^29^ ^]^ This suggested that the early stages of pseudotime represented a quiescent fibroblast phenotype, while the end indicated activated myofibroblasts. As fibroblasts in the KO group were particularly enriched in the late pseudotime clusters, Gene ontology (GO) enrichment analysis revealed that the late states were associated with extracellular matrix (ECM) organization and extracellular structure organization, further confirming the myofibroblast phenotype in KO cells (Figure 3F). Kyoto Encyclopedia of Genes and Genomes (KEGG) analysis was then performed to elucidate the underlying mechanisms. Cluster2 was enriched in pathways related to ECM‐receptor interaction and protein processing in the endoplasmic reticulum, while Cluster4 was enriched in pathways related to the Lysosome, phagosome, and protein processing in the endoplasmic reticulum (Figure 3G). Together, these findings reveal that AMFR deletion promotes cardiac fibroblast activation and impairs autophagy activity.

### 2.4 AMFR Deficiency Exacerbates Cardiac Fibrosis and Dysfunction Caused by MI

AMFR knockout (KO) mice were constructed using the CRISPR/Cas9 technology (Figure S4, Supporting Information). Echocardiography showed no noticeable differences in cardiac function between WT and AMFR KO mice under physiological conditions. H&E staining showed no significant differences in myocardial structure or arrangement between WT and AMFR KO mice (Figure S4, Supporting Information).

**Figure 4:**
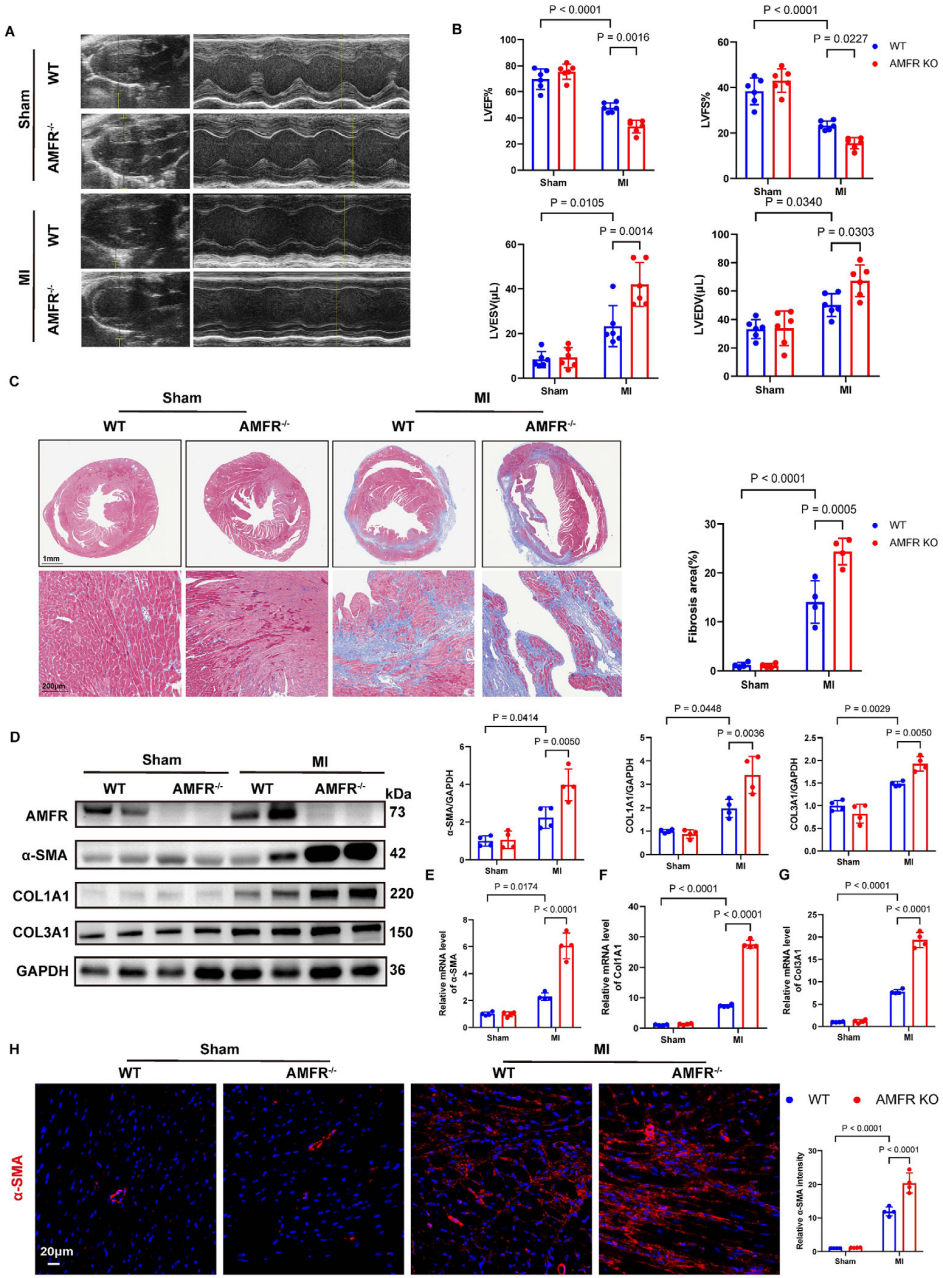
AMFR deficiency exacerbated cardiac fibrosis and dysfunction caused by MI. 8‐week‐old WT and AMFR knockout male mice were subjected to Sham or MI surgery, and heart tissues were harvested 2 weeks post‐surgery. A,B) Representative serial echocardiography and quantification of echocardiographic parameters. n = 6. C) Representative images and quantitative analyses of Masson's trichrome staining. n = 4. D) Representative Western blots and quantitative analyses of α‐SMA, COL1A1, and COL3A1 in heart tissues. n = 4. E–G) The relative mRNA levels of Acta2, Col1a1, and Col3a1 in heart tissues. n = 4. H) Representative images and quantitative analyses of immunofluorescence staining with α‐SMA (red) and DAPI (blue). n = 4. Data are presented as mean ± SD. The data were analyzed using two‐way ANOVA corrected by the post hoc Turkey's test. MI, myocardial infarction; LVEF, left ventricle ejection fraction; LVFS, left ventricle fractional shortening; LVEDV, left ventricle end diastolic volume; LVESV, left ventricle end systolic volume.

At day 1 post‐MI, no significant differences were observed between AMFR KO and WT mice (Figure S4, Supporting Information). However, by day 14 post‐MI, AMFR deficiency significantly worsened cardiac function, as evidenced by markedly declined left ventricle ejection fraction (LVEF%) and left ventricle fractional shortening (LVFS%) (**Figure** **4**A,B). Masson's trichrome staining further revealed that AMFR deficiency resulted in exacerbated cardiac fibrosis and increased collagen deposition at the infarct area compared to WT controls 14 days after MI (Figure 4C). Furthermore, AMFR deficiency significantly increased the MI‐induced upregulation of Col1a1, Col3a1, and α‐SMA at protein levels (Figure 4D) as well as mRNA levels (Figure 4E–G). Additionally, immunofluorescence staining showed that α‐SMA was significantly increased in the myocardium of AMFR KO mice post‐MI compared to WT littermates (Figure 4H). Collectively, these data elucidated that AMFR knockout exacerbated MI‐induced cardiac remodeling and dysfunction.

### 2.5 AMFR Overexpression Inhibits Fibroblast Proliferation, Migration, and Myofibroblast Differentiation

**Figure 5:**
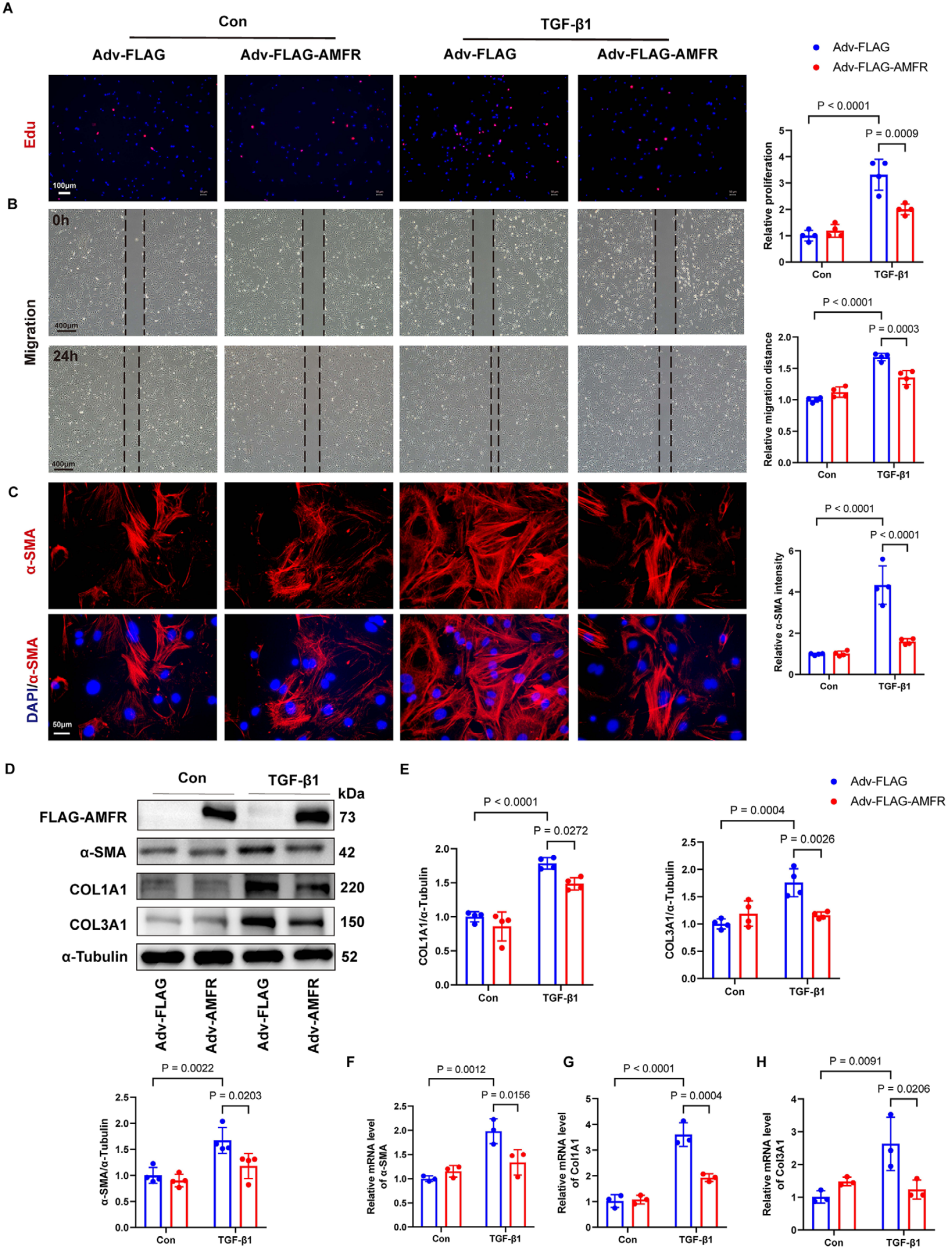
Overexpression of AMFR in cardiac fibroblasts inhibited proliferation, migration, and transdifferentiation of fibroblasts in vitro. NRCFs were isolated from 1 to 3‐day‐old neonatal Sprague‐Dawley rats and transfected with Adv‐AMFR or Adv‐Flag for 48 h before being treated with TGF‐β1(10 ng mL^−1^) for 24 h. A) Representative image and quantitative analyses showing EdU (red) localization with DAPI (blue). n = 4. B) Representative images and quantitative analyses of wound healing assays. n = 4. C) Representative images and quantitative analyses of immunofluorescence staining with α‐SMA (red) and DAPI (blue). n = 4. D,E) Representative Western blots and quantitative analyses of α‐SMA, COL1A1, and COL3A1 in NRCFs. n = 4. F–H) The relative mRNA levels of Acta2, Col1a1, and Col3a1 in NRCFs. All normalized to HPRT. n = 3. Data are presented as mean ± SD. The data were analyzed using two‐way ANOVA corrected by the post hoc Turkey's test. TGF‐β1, transforming growth factor‐β1; NRCFs, neonatal rat cardiac fibroblasts.

Adenoviral vectors (Adv‐AMFR) were used to overexpress AMFR in NRCFs (Figure S5, Supporting Information). Fibroblast proliferation and migration were assessed using EdU and wound healing assays, respectively. Compared to NRCFs with Adv‐Control, AMFR overexpression markedly inhibited fibroblast proliferation and migration in response to TGF‐β1 administration (**Figure** **5**A,B). Of note, α‐SMA fluorescence staining indicated that AMFR overexpression significantly inhibited TGF‐β1‐induced fibroblast‐to‐myofibroblast transdifferentiation (Figure 5C). Western blot analysis confirmed that AMFR overexpression downregulated the protein levels of α‐SMA, COL1A1, and COL3A1 in fibroblasts after TGF‐β1 administration (Figure 5D,E). Consistently, overexpression of AMFR also reduced the TGF‐β1‐induced upregulation of α‐SMA, Col1a1, and Col3a1 mRNA levels in fibroblasts (Figure 5F–H). Collectively, these results indicated that AMFR overexpression suppressed fibroblast proliferation, migration, and differentiation into myofibroblasts.

### 2.6 AMFR Deficiency Impairs ER‐phagy in Cardiac Fibroblasts

**Figure 6:**
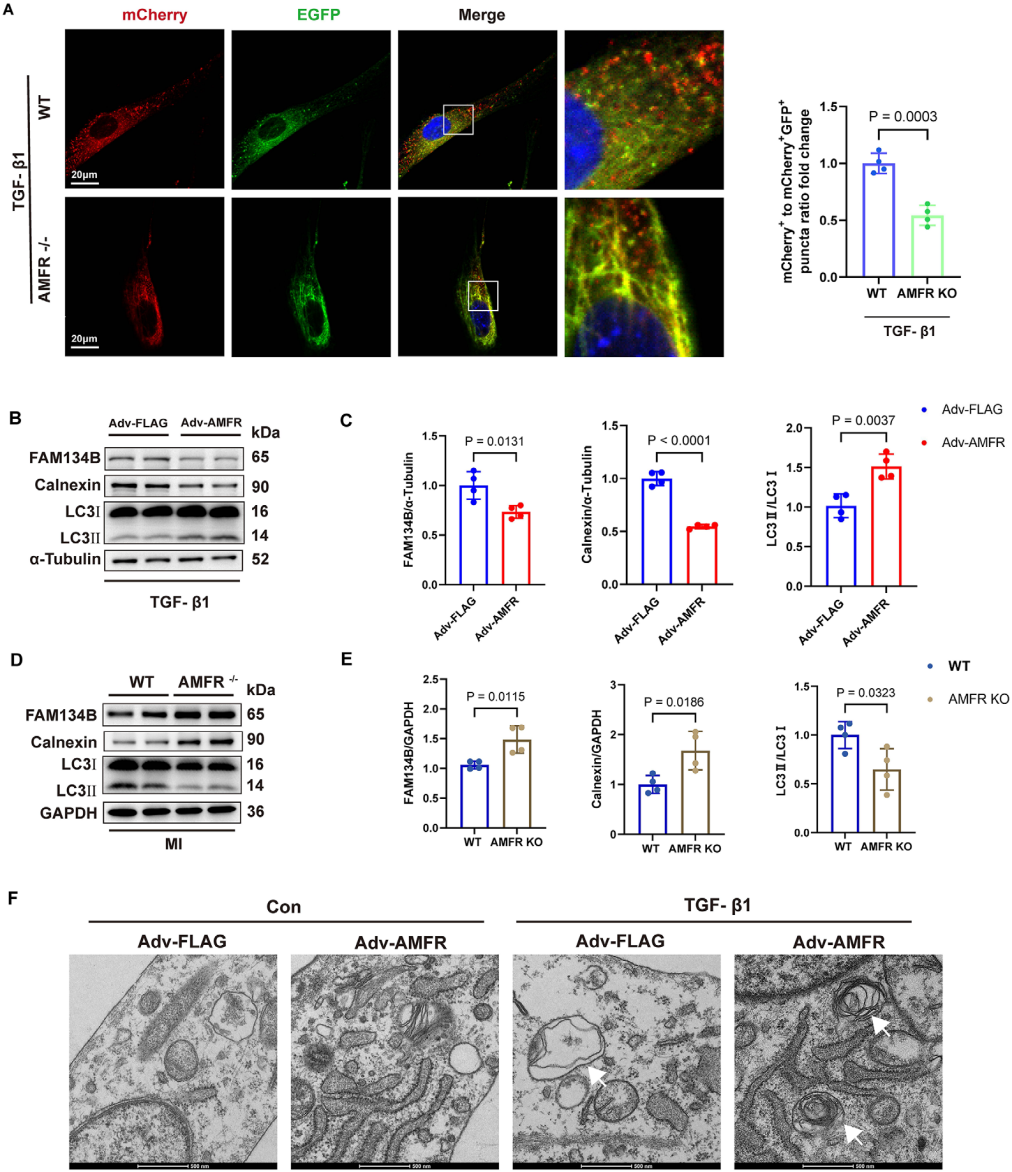
AMFR deficiency causes ER‐phagy defects in cardiac fibroblasts. A) ACFs isolated from WT or AMFR knockout mice transfected with Adv‐mCherry–GFP–FAM134B before being treated with TGF‐β1(10 ng mL^−1^). n = 4. B,C) Representative Western blots of FAM134B and Calnexin in NRCFs transfected with Adv‐AMFR or Adv‐Flag before being treated with TGF‐β1(10 ng mL^−1^). n = 4. D,E) Representative Western blots of FAM134B and Calnexin in heart tissues of WT and AMFR knockout mice 2 weeks post‐MI surgery. n = 4. F) Transmission electron microscopic imaging of NRCFs transfected with Adv‐AMFR or Adv‐Flag before being treated with PBS or TGF‐β1(10 ng mL^−1^). Data are presented as mean ± SD. The data shown in A, C, and E were analyzed using an unpaired t‐test. MI, myocardial infarction; TGF‐β1, transforming growth factor‐β1; ACFs, adult mouse primary cardiac fibroblasts; NRCFs, neonatal rat cardiac fibroblasts.

As FAM134B‐mediated ER‐phagy is activated in cardiac fibroblasts after TGF‐β1 stimulation (Figure 1), mCherry‐GFP‐FAM134B was employed to evaluate the role of AMFR in regulating ER‐phagy activity. After TGF‐β1 administration, mCherry signals in AMFR knockout fibroblasts were reduced compared to WT fibroblasts, demonstrating impaired ER‐phagy (**Figure** **6**A). Furthermore, Western blot analysis indicated that overexpression of AMFR decreased the protein level of FAM134B and ER marker Calnexin after TGF‐β1 administration (Figure 6B,C). Consistently, AMFR deficiency significantly increased the protein levels of FAM134B and Calnexin in heart tissues post‐MI (Figure 6D,E). Transmission electron microscope revealed that AMFR‐overexpressing fibroblasts contained massive autophagic vesicles and exhibited reduced ER enlargement and swelling (Figure 6F). Together, these findings suggest that AMFR plays a crucial role in regulating ER‐phagy in cardiac fibroblasts.

### 2.7 AMFR Ubiquitinates FAM134B and Promotes ER‐phagy to Suppress Cardiac Fibroblast Activation

**Figure 7:**
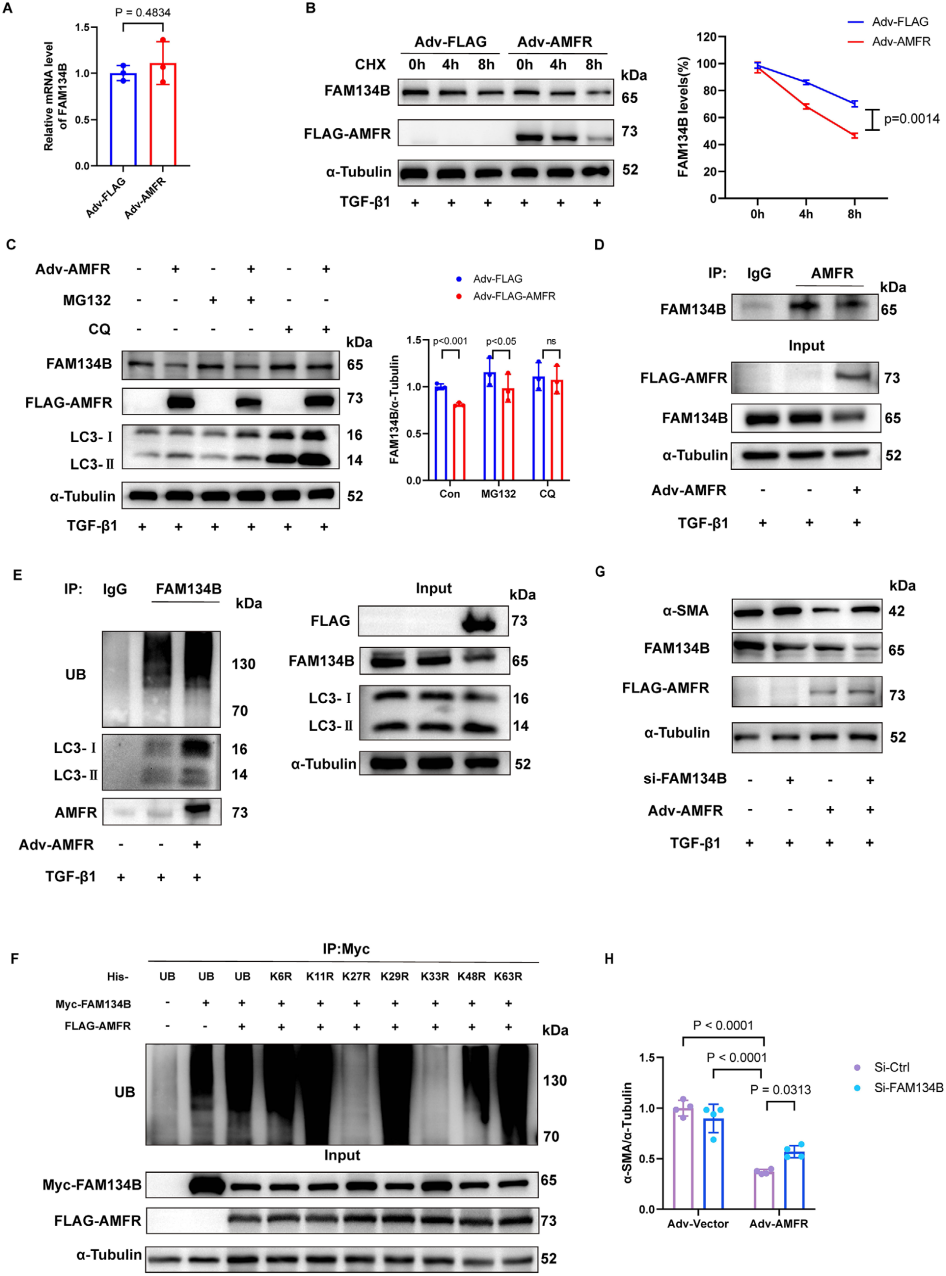
AMFR ubiquitinates FAM134B and promotes ER‐phagy to suppress cardiac fibroblast activation. A) The relative mRNA level of FAM134B in NRCFs transfected with Adv‐AMFR or Adv‐FLAG, followed by administration of TGF‐β1(10 ng mL^−1^), measured using RT‐qPCR analysis. n = 3. B) Western blot analysis of FAM134B in NRCFs transfected with Adv‐AMFR or Adv‐FLAG and incubated with cycloheximide (CHX) for the indicated times, followed by administration of TGF‐β1(10 ng mL^−1^). n = 3. C) NRCFs were transfected with Adv‐AMFR or Adv‐FLAG, then incubated with chloroquine (CQ) or MG132, followed by administration of TGF‐β1(10 ng mL^−1^) for 24 h. n = 3. D) NRCFs were transfected with Adv‐Flag or Adv‐AMFR for 48 h. Immunoprecipitation with the antibody against AMFR, followed by immunoblot with anti‐FAM134B antibody. IgG as a negative control. n = 3. E) NRCFs were transfected with Adv‐Flag or Adv‐AMFR for 48 h. Immunoprecipitation with the antibody against FAM134B, followed by immunoblot with anti‐AMFR, anti‐UB, and anti‐LC3 antibodies. IgG as a negative control. n = 3. F) HEK293T cells transduced with His‐Ub or His‐K6R‐UB, His‐K11R‐UB, His‐K27R‐UB, His‐K29R‐UB, His‐K33R‐UB, His‐K48R‐UB, His‐K63R‐UB, and MYC‐FAM134B, Flag‐AMFR vectors were immunoprecipitated with the antibody against MYC, and followed by immunoblot with anti‐UB antibody. IgG as a negative control. n = 3. G,H) Representative Western blots of α‐SMA in NRCFs transfected with or without Adv‐AMFR and siRNA‐FAM134B before being treated with TGF‐β1(10 ng mL^−1^). n = 4. Data are presented as mean ± SD. The data shown in A were analyzed using an unpaired t‐test. The data shown in B, C, and H were analyzed using two‐way ANOVA corrected by the post hoc Turkey's test. TGF‐β1, transforming growth factor‐β1; CHX, cycloheximide; CQ, chloroquine; NRCFs, neonatal rat cardiac fibroblasts; UB, ubiquitin.

Given the role of AMFR in promoting ER‐phagy, we sought to investigate its underlying mechanism and its impact on cardiac fibroblast activation. As depicted in Figure 6B, overexpression of AMFR significantly reduced FAM134B protein levels. Subsequently, we aimed to determine whether this effect occurred at the transcriptional or post‐translational level by measuring FAM134B mRNA levels following AMFR overexpression. The results revealed that no significant changes in the transcriptional level of FAM134B were observed after AMFR overexpression (**Figure** **7**A). AMFR overexpression decreased the protein level of FAM134B following cotreatment with a protein synthesis inhibitor, cycloheximide (CHX) (Figure 7B), indicating that AMFR may regulate the turnover of FAM134B post‐translationally.

Intracellular protein degradation is mainly mediated by the proteasomal or lysosomal pathway.^[^ ^30^ ^]^ Western blot analysis showed that overexpression of AMFR leads to the degradation of FAM134B, which can be reversed by the autophagy inhibitor chloroquine (CQ), but not by the proteasome inhibitor MG132 (Figure 7C), indicating that the degradation of FAM134B induced by AMFR is mediated through the autophagy pathway. Moreover, the Co‐IP assay confirmed the interaction between FAM134B and LC3 in NRCFs. We also examined the interaction between AMFR and other endoplasmic reticulum autophagy receptors in fibroblasts, and the results showed no significant interaction between AMFR and SEC62 as well as CCPG1 (Figure S6, Supporting Information).

As AMFR functions as an E3 ubiquitin ligase, we next investigated whether AMFR participates in the ubiquitination of FAM134B. We found that FAM134B ubiquitination was markedly increased in fibroblasts overexpressing AMFR (Figure 7D,E). As different types of polyubiquitin linkages mediate distinct biological functions, we expressed Myc‐tagged FAM134B in 293T cells together with various ubiquitin mutants (K6, K11, K27, K29, K33, K48, and K63), all of whose lysines were replaced with arginine. Notably, the K27R substitution and K33R substitution, rather than other point mutations, attenuated AMFR‐mediated ubiquitination of FAM134B (Figure 7F). To clarify which linkage (K27 or K33) predominates, HEK293T cells were transfected with His‐UB, His‐K27R‐UB, or His‐K33R‐UB, followed by treatment with chloroquine (CQ) to inhibit the degradation of autophagolysosome contents. Consistent with the previous results (Figure 7F), the K27R substitution and K33R substitution attenuated AMFR‐mediated ubiquitination of FAM134B. Chloroquine treatment increases the detected levels of FAM134B ubiquitination across all groups by inhibiting the degradation of ubiquitinated FAM134B. Notably, the ubiquitination levels in the K33R substitution group showed a more pronounced increase compared to the K27R group (Figure S7, Supporting Information), supporting the dominance of K27‐linked ubiquitination. Importantly, knockdown of FAM134B by siRNA abolished AMFR‐mediated inhibition of α‐SMA level in cardiac fibroblasts (Figure 7G), providing further evidence that the inhibitory effects of AMFR on fibroblasts are, at least in part, exerted by the promotion of ER‐phagy. Collectively, these data prove that AMFR ubiquitinates FAM134B and targets it for degradation via the autophagy pathway, which is crucial for AMFR‐mediated suppression of cardiac fibroblast activation.

### 2.8 AMFR Inhibits mTORC1 Signaling in a FAM134B‐Dependent Manner

**Figure 8:**
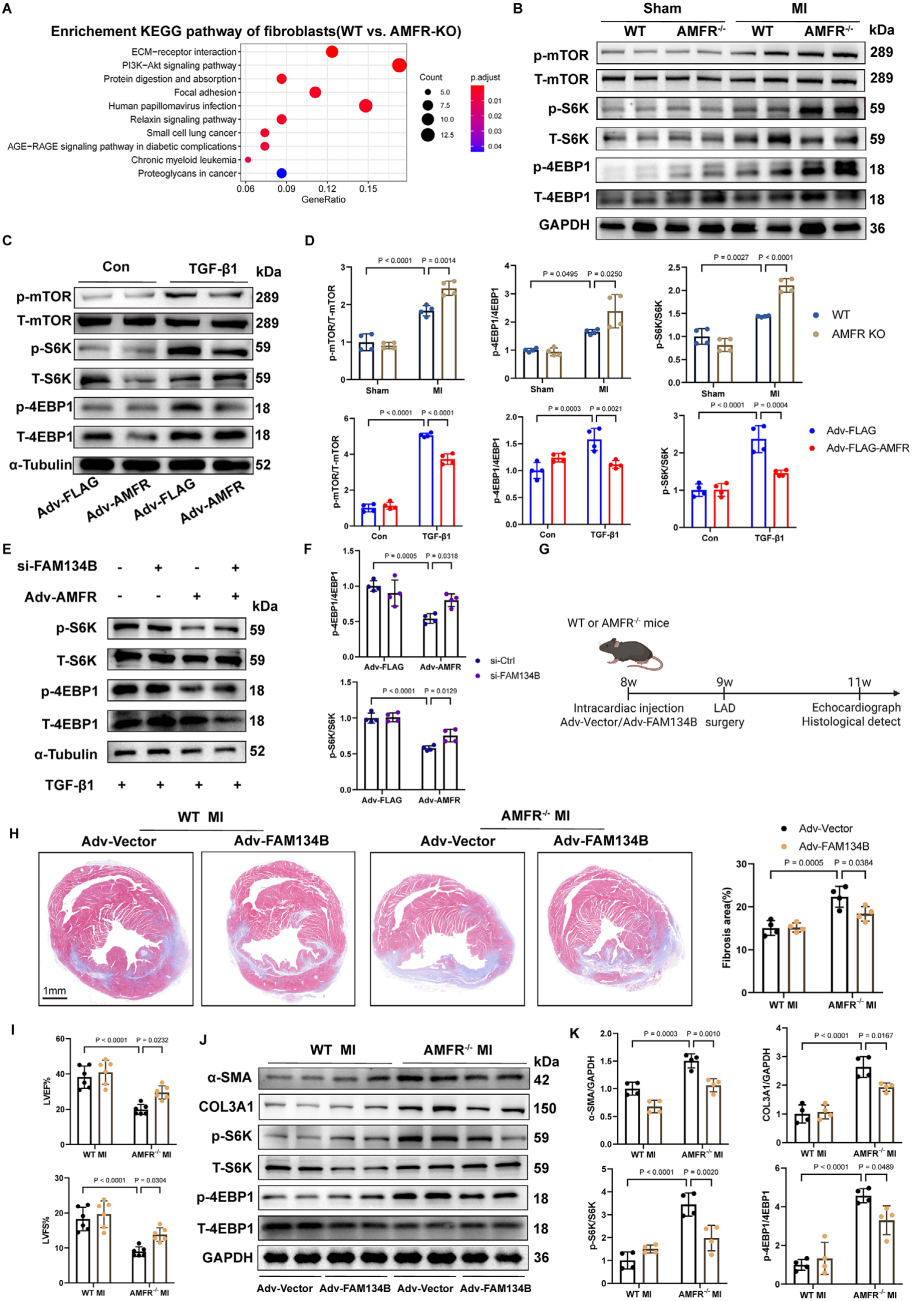
AMFR inhibits the mTORC1 signaling in a FAM134B‐dependent manner. A) KEGG analysis identified changed pathways in the cardiac fibroblasts isolated from WT or AMFR‐knockout mice post‐MI. B) Representative Western blots of p‐mTOR, p‐S6K, and p‐4E‐BP1 in heart tissues of WT and AMFR KO mice at 2 weeks post‐MI. n = 4. C) Representative Western blots of p‐mTOR, p‐S6K, and p‐4E‐BP1 in NRCFs transfected with Adv‐AMFR or Adv‐Flag before being treated with TGF‐β1(10 ng mL^−1^). n = 4. D) Representative statistical result of B and C. E,F) Representative Western blots and statistical result of p‐4EBP1 and p‐S6K in NRCFs transfected with or without Adv‐AMFR and siRNA‐FAM134B before being treated with TGF‐β1(10 ng mL^−1^). n = 4. G) Schematic diagram of the experimental model. 8‐week‐old WT or AMFR‐knockout male mice were transfected with Adv‐FAM134B or Adv‐Vector via intracardiac injection for one week before being subjected to Sham or MI surgery, and heart tissues were harvested 2 weeks post‐surgery. H) Masson's trichrome staining and the corresponding statistical result. n = 4. I) Quantification of echocardiographic parameters of ejection fraction (EF%) and fractional shortening (FS%). n = 6. J,K) Representative Western blots and statistical results of α‐SMA, COL3A1, p‐4EBP1, and p‐S6K in heart tissues. n = 4. Data are presented as mean ± SD. The data in D, F, H, I, and K were analyzed using two‐way ANOVA corrected by the post hoc Turkey's test. KEGG, Kyoto Encyclopedia of Genes and Genomes; MI, myocardial infarction; TGF‐β1, transforming growth factor‐β1; NRCFs, neonatal rat cardiac fibroblasts; LVEF, left ventricle ejection fraction; LVFS, left ventricle fractional shortening.

To fully understand the molecular mechanisms underlying AMFR‐mediated cardiac remodeling, we next identified the pathway most significantly affected by AMFR. KEGG pathway enrichment analysis was used to identify the biological pathways involved in cardiac fibroblasts from WT and AMFR knockout mice after MI. The results revealed that the PI3K‐AKT signaling pathway was significantly enriched (**Figure** **8**A). As the TGF‐β/SMAD pathway is a canonical pathway for the activation of fibroblasts, we detected the phosphorylation levels of SMAD2 and SMAD3. Our findings revealed that AMFR did not affect the phosphorylation levels of SMAD2 or SMAD3 (Figure S8, Supporting Information). Since mTORC1 is downstream of PI3K‐AKT signaling pathway known to promote fibrosis,^[^ ^31^, ^32^ ^]^ we then examined phosphorylation of mTOR and key effectors of mTORC1, including S6K1 and 4E‐BP1.^[^ ^33^, ^34^, ^35^ ^]^ The results confirmed that AMFR deficiency significantly increased the MI‐induced upregulation of p‐mTOR, p‐P70 S6K, and p‐4EBP1 protein levels (Figure 8B). In NRCFs, overexpression of AMFR inhibited the mTORC1 signaling pathway after TGF‐β1 administration, as evidenced by decreased p‐mTOR, p‐S6K, and p‐4E‐BP1 levels (Figure 8C,D). To verify whether the antifibrotic effect of AMFR is directly mediated through the mTORC1 pathway, we employed rapamycin to inhibit the mTORC1 pathway. siRNA‐mediated AMFR silencing enhanced α‐SMA protein levels, but co‐treatment with rapamycin effectively abrogated the pro‐fibrotic effect induced by si‐AMFR (Figure S8, Supporting Information).

Given the exclusive functional specificity of FAM134B in mediating ER‐phagy, we performed FAM134B knockdown to attenuate ER‐phagy. The results showed that knockdown of FAM134B by siRNA abolished AMFR‐mediated inhibition of p‐S6K and p‐4E‐BP1 levels in the cardiac fibroblasts (Figure 8E,F). To further explore the in vivo relevance, WT and AMFR KO mice were transfected with Adv‐Flag or Adv‐FAM134B prior to MI construction (Figure 8G). Transfection with Adv‐FAM134B in the myocardium did not affect cardiac function or fibrosis in sham mice at 2 weeks (Figure S9, Supporting Information). Masson's trichrome revealed that overexpression of FAM134B significantly reversed the increase of fibrosis in AMFR knockout mice (Figure 8H). In MI mice, echocardiographic analysis showed that overexpression of FAM134B alleviated the decreased EF% and FS% in mice caused by AMFR deficiency (Figure 8I). Western blot analysis revealed that α‐SMA, COL3A1, p‐S6K, and p‐4E‐BP1 levels were significantly increased in the heart tissues of AMFR KO mice, but were reversed after overexpressing FAM134B (Figure 8J,K). Accordingly, these findings suggest that AMFR promotes ER‐phagy, thereby contributing to the inhibition of mTORC1 signaling and suppressing fibroblast activation.

### 2.9 Fibroblast‐Specific AMFR Overexpression Attenuates Cardiac Fibrosis and Improves Cardiac Function Post‐MI

**Figure 9:**
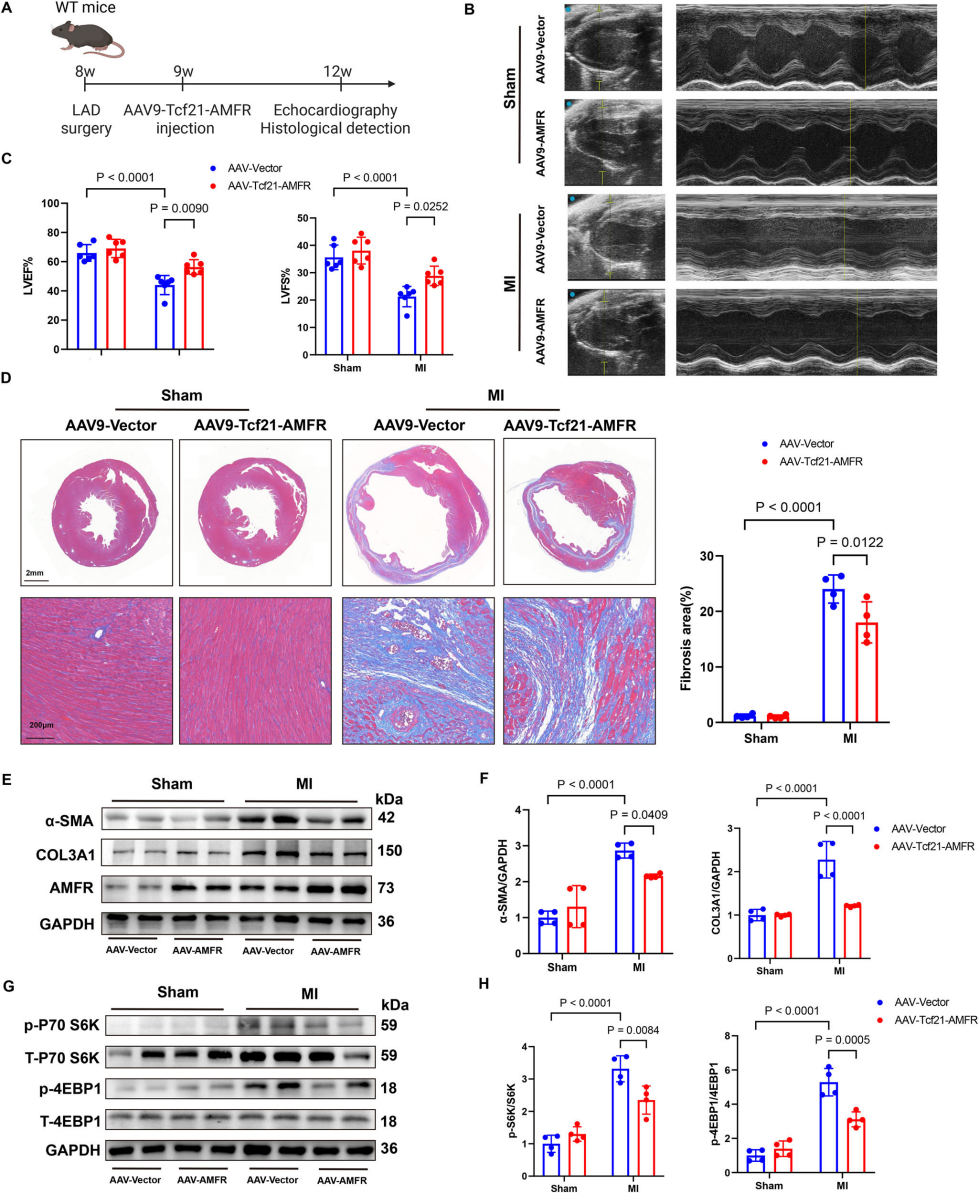
Fibroblast‐specific AMFR overexpression attenuated cardiac fibrosis and improved cardiac dysfunction post‐MI. 8‐week‐old WT male mice were transfected with AAV9‐Tcf21‐AMFR or AAV‐Vector via tail vein injection one week after being subjected to Sham or MI surgery, and heart tissues were harvested 4 weeks post‐surgery. A) Treatment regimen. B,C) Representative serial echocardiography and quantification of echocardiographic parameters of ejection fraction (EF%) and fractional shortening (FS%). n = 6. D) Masson's trichrome staining and the corresponding statistical result of fibrosis. n = 4. E,F) Representative Western blots and statistical results of α‐SMA and COL3A1 in heart tissues. n = 4. G,H) Representative Western blots and statistical results of p‐S6K and p‐4E‐BP1 in heart tissues. n = 4. Data are presented as mean ± SD. The data were analyzed using two‐way ANOVA corrected by the post hoc Turkey's test. MI, myocardial infarction; LVEF, left ventricle ejection fraction; LVFS, left ventricle fractional shortening.

To investigate the therapeutic potential of AMFR in cardiac remodeling, we established fibroblast‐specific AMFR‐overexpressing mice by administering rAAV9‐Tcf21‐AMFR via tail vein injection (**Figure** **9**A). Western blot confirmed successful fibroblast‐specific AMFR overexpression in mice (Figure 9E). Echocardiographic analysis revealed that AMFR overexpression in fibroblasts mitigated cardiac dysfunction induced by MI, indicated by increased EF% and FS% (Figure 9B,C). Masson's trichrome staining showed that collagen deposition caused by MI was significantly reduced in the AMFR‐overexpressing mice hearts (Figure 9D). Western blot analysis revealed that AMFR overexpression attenuated MI‐induced increases in α‐SMA, COL3A1, p‐S6K and p‐4E‐BP1 levels (Figure 9E–H). Consequently, the in vivo experiments proved that fibroblast‐specific AMFR overexpression is a potential target to inhibit the progression of cardiac fibrosis post‐MI.

## 3 Discussion

**Figure 10:**
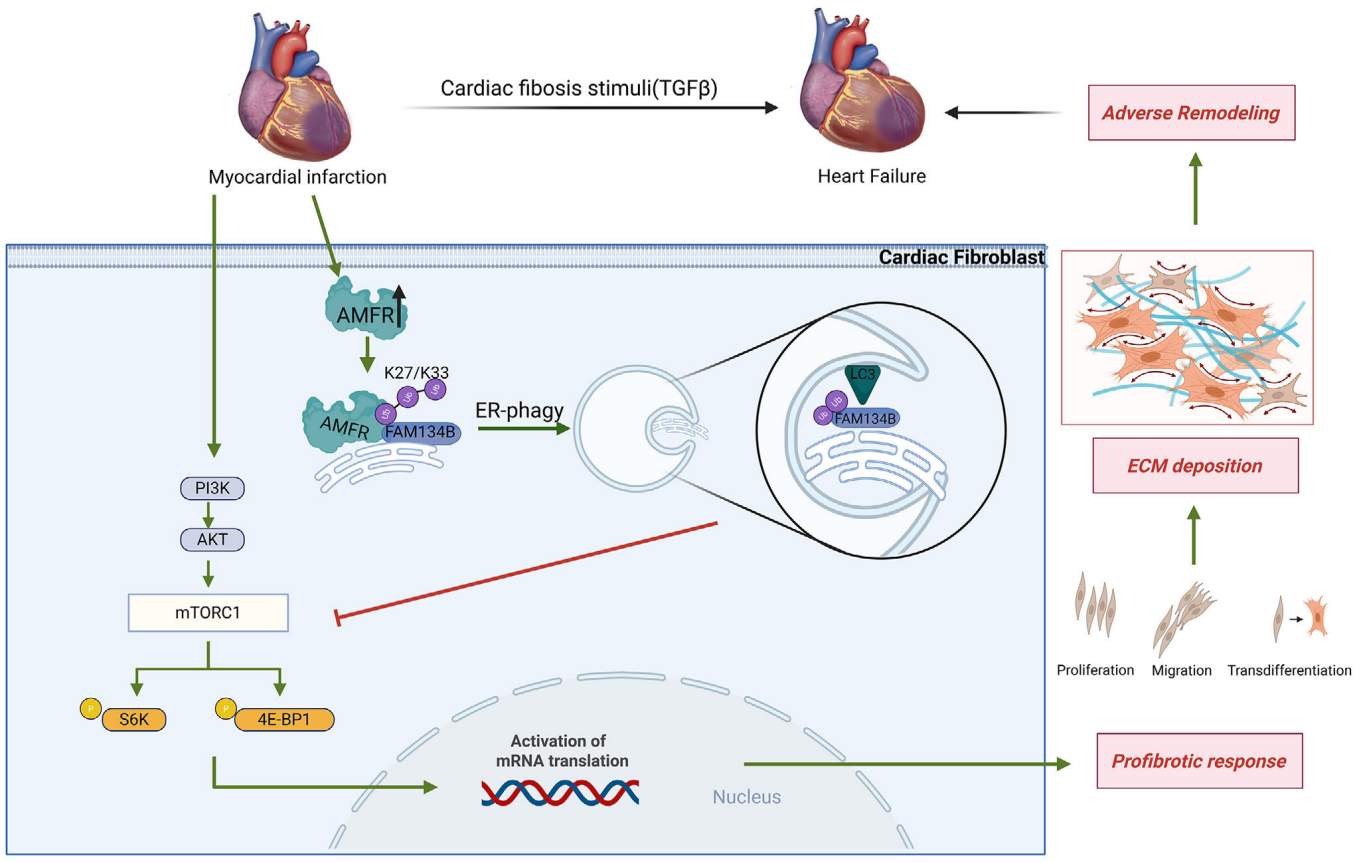
Schematic depiction of the regulatory mechanism in which AMFR catalyzes K27‐linked (predominant) and K33‐linked ubiquitination of FAM134B and promotes ER‐phagy activity, thereby inhibiting the mTORC1 signaling pathway during the progression of fibrosis post‐MI.

In this study, we have elucidated the crucial role of AMFR‐mediated ER‐phagy in cardiac remodeling following MI. Our findings highlight that AMFR targets the ubiquitination of the ER‐phagy receptor FAM134B, facilitating ER‐phagy and subsequent inhibition of mTORC1 signaling in cardiac fibroblasts. This discovery not only uncovers the role of ER‐phagy in regulating cardiac fibroblasts but also unveils the AMFR/FAM134B/mTORC1 axis as a novel mechanism during cardiac remodeling, providing potential therapeutic targets for the prevention of heart failure post‐MI (**Figure** **10**).

Cardiac fibrosis acts as a double‐edged sword in the context of myocardial infarction repair. Initial fibrosis at the early stage post‐MI is essential for preventing ventricular rupture and maintaining cardiac integrity.^[^ ^36^ ^]^ Our previous study revealed that Cthrc1 deficiency attenuated fibrosis repair at the early stage post‐MI, leading to increased mortality attributable to cardiac rupture.^[^ ^37^ ^]^ However, unrestrained or excessive activation of fibroblasts can drive pathological fibrosis. Timely activation and suppression of pro‐fibrotic signals are critical to prevent adverse remodeling and cardiac dysfunction.^[^ ^38^ ^]^ Here, we found that the AMFR/FAM134B/mTORC1 axis is pivotal in regulating cardiac fibrosis post‐MI.

After myocardial infarction, the level of autophagy exhibits dynamic changes and plays an important role in myocardial repair and remodeling.^[^ ^39^ ^]^ During both the subacute and chronic stages (1 and 3 weeks postinfarction, respectively), autophagy was found to be activated in mice subjected to MI surgery. Moreover, the activation of autophagy attenuates cardiac remodeling and improves cardiac function.^[^ ^40^, ^41^ ^]^ In this study, we discover that ER‐phagy is activated in fibrotic mouse‐heart tissues after myocardial infarction and in cardiac fibroblasts stimulated by TGF‐β1(Figure 1). ER‐phagy is the selective autophagy mediated by specific receptors, contributing to ER homeostasis and alleviating ER stress.^[^ ^7^ ^]^ So far, researchers have found ER‐phagy is associated with various human disorders such as neurological disorders, cancer, and infections.^[^ ^8^, ^42^, ^43^ ^]^ Despite this, reports on the role of ER‐phagy in cardiovascular diseases, including myocardial infarction, are still limited at present. It has been found to alleviate anthracycline‐induced cardiotoxicity through CCPG1‐mediated signaling[17]. An increasing number of studies have demonstrated that inhibiting endoplasmic reticulum stress or promoting autophagy are strategies for the prevention and treatment of fibrosis.^[^ ^44^, ^45^ ^]^ However, the function of the selective ER‐phagy has not been explored in the context of fibroblast activation nor in fibrosis diseases. Our investigation reveals significant ER‐phagy activation in both fibrotic myocardium from MI‐induced mouse models and TGF‐β1‐stimulated cardiac fibroblasts, with FAM134B showing the most pronounced upregulation among ER‐phagy receptors. These findings highlight the potential significance of ER‐phagy in regulating fibrotic remodeling post‐MI, underscoring the critical involvement of ER quality control pathways in MI pathophysiology.

The first discovered mammalian ER‐phagy receptor, FAM134B (also known as RETREG1), was initially identified as an oncogene in esophageal squamous cell carcinoma. Over the past few decades, the potent biological functions of FAM134B have been gradually revealed. A large body of evidence has shown that its dysfunction is closely related to a variety of pathological processes, including neuropathy, viral replication, inflammation, and cancer.^[^ ^46^ ^]^ Building upon previous findings that AMFR catalyzes FAM134B ubiquitination,^[^ ^20^ ^]^ our study advances this understanding by identifying K27‐linked (predominant) and K33‐linked ubiquitination of FAM134B by AMFR in cardiac fibroblasts as a critical mechanism driving ER‐phagy activation. Notably, prior work by D. Mookherjee et al.^[^ ^47^ ^]^ demonstrated that FAM134B mediates autophagosomal degradation of both AMFR and OPA1, a mitochondrial fusion protein, thereby maintaining ER‐mitochondria homeostasis. This mechanistic insight at the cellular scale now calls for extended investigation into how such degradation pathways might have an effect at the organ level and contribute to pathophysiological cascades. Our findings of ubiquitination‐dependent regulation align with the established role of FAM134B in coordinating ER and mitochondrial quality control. In our study, we extended the role of ER‐phagy to the realm of fibroblast activation and confirmed that AMFR suppressed cardiac fibrosis post‐MI through FAM134B‐mediated ER‐phagy. These results position AMFR not merely as a ubiquitination enzyme but as a central orchestrator of ER quality control and fibroblast activation through dynamic interactions with FAM134B.

Substantial evidence has verified the effects of AMFR in regulating invasion and metastasis of tumor cells,^[^ ^48^ ^]^ inflammatory responses,^[^ ^21^, ^22^ ^]^ innate immunity,^[^ ^23^ ^]^ allergic asthma,^[^ ^24^ ^]^ and differentiation of lung fibroblasts.^[^ ^49^ ^]^ Notably, previous research has highlighted the pro‐fibrotic role of AMFR in pulmonary contexts, where it promotes lung fibroblast differentiation via the CCL1‐AMFR‐SPRY1 axis, activating the Ras‐ERK pathway.^[^ ^49^ ^]^ Paradoxically, our study unveils its previously unrecognized cardioprotective role in suppressing cardiac fibrosis through a fundamentally distinct mechanism. Our study identifies AMFR as a critical suppressor of cardiac fibrosis, evidenced by exacerbated fibrotic remodeling and cardiac dysfunction post‐MI in global AMFR knockout mice, and further supported by pseudotemporal trajectory analysis revealing enhanced myofibroblast differentiation in AMFR deficient fibroblasts. This opposite function likely stems from differences in organ‐specific microenvironments, fibroblast types, and downstream signaling networks. Fibroblasts are diverse in function and transcription across and within organs. Despite sharing similar properties and responding to many of the same signal transduction pathways, tissue specific fibroblast functions and lineages exist to support the developmental, homeostatic, and repair needs of specific organs. During development, cardiac fibroblasts form from epicardial and endocardial epithelial cells via epithelial‐to‐mesenchymal transition (EMT) and endothelial‐to‐mesenchymal transition (EndMT), respectively. This is in contrast to the fibroblasts of other organs, such as the lung, that arise from progressive differentiation of mesenchymal progenitors.^[^ ^50^, ^51^, ^52^ ^]^ Therefore, the same protein or molecule may exert entirely different effects in fibroblasts of different organs. This “paradoxical” phenomenon suggests that therapies targeting AMFR should be precisely regulated in conjunction with organ‐specific mechanisms.

The TGF‐β/Smad signaling pathway and PI3K/AKT/mTORC1 signaling pathway are the classic pathways mediating tissue fibrosis.^[^ ^53^ ^]^ KEGG analysis showed that the PI3K‐AKT signaling pathway was significantly enriched in AMFR knockout fibroblast post‐MI. mTORC1 phosphorylates two key effectors of translation, S6K1 and 4E‐BP1, to conduct protein synthesis, which promotes cell proliferation and fibrotic response.^[^ ^33^, ^34^, ^35^ ^]^ Our findings revealed that AMFR inhibits phosphorylation of S6K1 and 4E‐BP1 both in vitro and in vivo experiments rather than SMAD2 and SMAD3. Compared with other known fibrosis regulatory pathways (such as TGF‐β/Smad, Wnt/β‐catenin, etc.), mTORC1 integrates nutrient and energy signals, directly regulating cellular anabolism and the synthesis of extracellular matrix proteins.^[^ ^54^ ^]^ The specificity of AMFR in inhibiting the mTORC1 pathway highlights its intricate and unique role in modulating autophagy and protein synthesis. mTORC1 serves as a pivotal regulator throughout the autophagic cascade—suppressing autophagosome initiation through Atg13/ULK1 phosphorylation and TFEB inactivation.^[^ ^55^ ^]^ In turn, lysosome‐derived amino acids (leucine and arginine) and also other nutrients (glucose‐DHAP, cholesterol, SAM) can partially reactivate Rag GTPase–nutrient signaling upstream of mTORC1.^[^ ^56^, ^57^ ^]^ This indicates that there is a mutual regulatory relationship between mTORC1 and autophagy. Our research has uncovered that AMFR promotes ER‐phagy (Figure 6) while simultaneously inhibiting the mTORC1 pathway (Figure 8), which is not contradictory to the established theory that mTORC1 inhibits autophagy. However, this presents a critical mechanistic dilemma: who serves as the upstream regulator—ER‐phagy or the mTORC1 pathway? By conducting in vitro FAM134B knockdown and in vivo FAM134B overexpression experiments, we have demonstrated that ER‐phagy is indeed upstream of the mTORC1 pathway. This epistatic relationship definitively positions ER‐phagy upstream of mTORC1 in cardiac fibroblasts, establishing a novel regulatory axis where AMFR/FAM134B‐mediated ER‐phagy restrains mTORC1‐driven profibrotic signaling. Accordingly, we claimed that AMFR promotes the ER‐phagy activity, contributing to the inhibition of the mTORC1 signaling associated with fibroblast activation. For the first time, we have demonstrated that ER‐phagy can regulate mTORC1 signaling, suggesting that AMFR/FAM134B‐mediated ER‐phagy is a pivotal mechanism in the regulation of fibroblast activation.

The mechanisms by which AMFR affects S6K and 4E‐BP1 through ER‐phagy are likely highly complex and still need further investigation. The mTORC1 complex comprises mTOR, Raptor, mLST8, and two inhibitory subunits, PRAS40 and DEPTOR. Traditionally, mTORC1 is recruited to the lysosomal surface and activated. This process is mediated by the Rag GTPase dimer, which is anchored to the lysosome through its interaction with the Ragulator complex.^[^ ^58^, ^59^, ^60^, ^61^ ^]^ However, a recent study has found that mTORC1 can also be activated outside of the lysosome,^[^ ^62^ ^]^ which implies that mTORC1 may be affected during the early stages of ER‐phagy, rather than being influenced only on the lysosomal surface. Thus, ER‐phagy may inhibit the mTORC1 pathway by affecting the degradation of mTORC1 components, the localization and activation states of mTORC1 in lysosomal and non‐lysosomal compartments, and the regulatory effects of Rheb on mTORC1. However, in the process of investigating the mechanisms through which ER‐phagy inhibits mTORC1, our study confronts two primary limitations. First, there is a gap in understanding the activation mechanisms of non‐lysosomal mTORC1; Second, research methods, such as lysosomal immunoprecipitation and Immuno‐Electron microscope, serve as significant technical bottlenecks. These limitations prevent us from directly exploring the specific molecular mechanisms by which ER‐phagy inhibits mTORC1 now.

Our research indicates that AMFR‐catalyzed K27‐and K33‐linked ubiquitination of FAM134B is a key mechanism in promoting ER‐phagy in fibroblasts. However, this is unlikely to be the sole mechanism. Other E3 ubiquitin ligases, such as TRIM13, have been shown to recruit LC3 via self‐ubiquitination,^[^ ^63^ ^]^ potentially modulating ER‐phagy under diverse stress conditions. Additionally, non‐canonical ubiquitination types or alternative post‐translational modifications, such as phosphorylation or ufmylation,^[^ ^64^, ^65^, ^66^ ^]^ might fine‐tune FAM134B activity or recruit alternative ER‐phagy receptors like SEC62 or CCPG1. Thus, while AMFR‐mediated ubiquitination is pivotal, the ER‐phagy network likely operates through redundant, tissue‐specific mechanisms to ensure robust stress adaptation.

Our research identifies the AMFR/FAM134B/mTORC1 axis as a critical regulator of cardiac fibrosis, primarily through its role in modulating ER‐phagy. AMFR and FAM134B are expressed ubiquitously across tissues. Meanwhile, mTORC1 plays a crucial regulatory role in various organs, involving multiple aspects such as cell growth, proliferation, metabolism, and stress responses.^[^ ^67^ ^]^ mTORC1 has been reported to regulate skin wound healing, promote renal fibrosis, et al.^[^ ^68^, ^69^ ^]^ Thus, targeting the AMFR/FAM134B/mTORC1 signaling axis to develop antifibrotic therapies represents a promising therapeutic strategy worthy of in‐depth investigation. Key research directions include developing small–molecule agonists or inhibitors targeting AMFR or the ER–phagy receptors to modulate the critical nodes of the axis, exploring AAV‐mediated gene therapy to regulate the expression of key genes such as AMFR or FAM134B, and utilizing biologics like antibodies targeting downstream proteins of mTORC1 to intervene in downstream functions. However, the field faces formidable challenges. The primary difficulty is that mTORC1 regulates a wide range of fundamental physiological processes,^[^ ^67^ ^]^ so drugs targeting this axis need to be highly selective to avoid side effects. It is essential to achieve tissue–and subcellular–specific drug delivery (e.g., targeting cardiac fibroblasts) to prevent off–target effects. Moreover, it is crucial to thoroughly elucidate the regulatory mechanisms of this axis in fibrosis of other organs and in other diseases (such as cancer, metabolic diseases, and neurodegenerative diseases) to provide a theoretical basis for precise intervention and to facilitate the safe clinical translation of these therapeutic strategies.

There are some limitations in this study. First, the detection of ER‐phagy flux within tissues remains challenging, which complicates our understanding of its role during MI. Nevertheless, through in vivo immunofluorescence co‐localization experiments, we have preliminarily validated the role of AMFR‐mediated ER‐phagy in the context of myocardial infarction. Second, while our study confirmed that FAM134B‐mediated ER‐phagy is essential to the inhibition of cardiac fibroblasts, the effects of other ER‐phagy receptors, such as CCPG1 and Sec62, which are also increased after TGF‐β1 administration, remain unclear. Different ER‐phagy receptors share the same ligands (such as LC3/GABARAP) or upstream signaling hubs (such as the ULK1 complex).^[^ ^8^ ^]^ Therefore, it cannot be ruled out that SEC62 and CCPG1 may compensate for the functions of FAM134B when it is downregulated. Future research should verify these mechanisms and their dysregulation in related diseases. Third, overexpression of FAM134B activated ER‐phagy activity in AMFR KO mice, suggesting that other ligands may also activate FAM134B and enhance ER‐phagy activity, which is necessary to clarify in the future. Fourth, our study lacks validation using human heart samples. Future research should conduct relevant experiments to enhance its clinical translational value. Lastly, although we demonstrated that AMFR inhibited the mTORC1 pathway, the specific protein targets involved in this process need further investigation. Additional molecular experiments and animal studies are required to fully elucidate the role and mechanisms of ER‐phagy in myocardial infarction.

## 4 Conclusion

This study uncovers a novel association between myocardial fibrosis and AMFR/FAM134B‐mediated ER‐phagy. By catalyzing FAM134B ubiquitination and activating ER‐phagy, AMFR alleviates progressive fibrosis and cardiac dysfunction by inhibiting the mTORC1 pathway. Consequently, our findings underscore the essential role of AMFR‐driven ER‐phagy in mitigating the progression of fibrotic responses, offering a potential therapeutic target for preventing heart failure post‐MI.

## 5 Experimental Section

### Experimental Animals

*Amfr* conventional knockout (*Amfr^−/−^*) mice were generated by Cyagen Bioscience Inc. (Guangzhou, China), while wild‐type (WT) C57BL/6 mice were purchased from Beijing Vital River Laboratory Animal Technology Co., Ltd. (Beijing, China). All animals were housed in standard conditions, with 12 h light/dark cycles, constant temperature, and ad libitum access to food and water throughout the experiments. All animal experiments were approved by The Animal Care And Use Committee of Shanghai Jiao Tong University Affiliated Sixth People's Hospital (Permit Number: DWLL2025‐0055), in accordance with the Guide for the Care and Use of Laboratory Animals established by the US National Institutes of Health (NIH Publication, 8th Edition, 2011).

Genotyping of all animals was carried out by PCR amplification of DNA extracted from mouse tail samples to confirm the knockout of *Amfr*. The genotyping strategies are shown in Table S2 (Supporting Information).

### Mice Model of Myocardial Infarction

Male mice 8 weeks old were anesthetized using 1.5% isoflurane with oxygen at a flow rate of 0.5 L min^−1^ via mechanical ventilation. Myocardial infarction was conducted by ligation of the left anterior descending (LAD) coronary artery with a 6‐0 prolene suture, while sham‐operated mice underwent the same procedure without LAD occlusion. After the indicated time, the mice were euthanized by the same anesthetics to obtain their heart samples for corresponding analyses. Subsequently, heart samples were either flash‐frozen in liquid nitrogen and stored at −80 °C for further RNA and protein analysis or fixed in a PBS solution containing 4% paraformaldehyde for histochemical analysis.

### Echocardiography

The assessment of cardiac functions was conducted utilizing the Vevo 2100 High‐Resolution Micro‐Ultrasound System (FUJIFILM Visual Sonics Inc.). Mice were subjected to anesthesia via a combination of isoflurane (1.5%) and oxygen (0.5 L min^−1^) and were placed in a supine position. B‐mode echocardiography was employed to guide the acquisition of left ventricular M‐mode images at the papillary muscle level in a long‐axis orientation. This approach enabled the quantification of key parameters, including ejection fraction (EF%) and fractional shortening (FS%). To ensure unbiased results, these measurements were performed by an investigator blinded to the experimental conditions and were averaged across five consecutive cardiac cycles.

### Construction of Adenovirus, Adeno‐Associated Virus, and Plasmid

Adenovirus (Adv) was packaged by OBiO (Shanghai, China). Adeno‐associated virus (AAV) was packaged by WZ Biosciences Inc. (Shandong, China). AAV‐RFP‐GFP‐KDEL and Adv‐mCherry‐GFP‐FAM134B were used to detect ER‐phagy flux. Adv‐AMFR and Adv‐FAM134B were used to manipulate the expression of AMFR and FAM134B in cultured fibroblasts and in mouse hearts, respectively. The heart‐specific Tcf21 promoter was utilized in AAV9 construction to drive AMFR overexpression specifically in fibroblasts. Mouse expression plasmids encoding UB or the corresponding UB mutant were produced and purchased from Miaoling (Wuhan, China).

### Adenovirus and Adeno‐Associated Virus Mediated Gene Transfection In Vivo

To establish a heart‐overexpressed FAM134B animal model, the cardiac adenoviral gene transfer approach was employed. The adenoviral in vivo delivery method was conducted as previously described.^[^ ^70^, ^71^ ^]^ In brief, a 20 µL solution of adenovirus containing 1 × 10^9 plaque‐forming units (pfu) of either Adv‐Flag or Adv‐FAM134B was delivered from the apex of the left ventricle to the aortic root. A tourniquet was placed around the aorta and the pulmonary artery at a site distal to the tip of the catheter, followed by the injection of the solution. The tourniquet was held in place for 10 s while the heart pumped against a closed system and then released. To examine the transduction efficiency, the hearts were harvested and sectioned two weeks after transduction, and FAM134B expression was detected by Western blots. Hearts were transduced with adenovirus, followed by MI surgery, and were harvested 2 weeks after surgery.

To establish a fibroblasts‐specific overexpressed FAM134B animal model, AAV9.Tcf21. Flag or AAV9. Tcf21.AMFR (3 × 10^11^ v.g./mouse) was administered through tail vein injection to eight‐week‐old male wild‐type mice one week after the surgical procedures. The hearts were harvested and sectioned three weeks after transduction.

### Cell Culture and Gene Transfer

Neonatal rat ventricular fibroblasts (NRCFs) were isolated from 1 to 3‐day‐old neonatal Sprague‐Dawley rats following established procedures.^[^ ^32^ ^]^ The cells were plated at a density of 10^6 cells mL^−1^ and cultured in Dulbecco's Modified Eagle Medium (DMEM, Gibco, USA) supplemented with 10% Fetal Bovine Serum (FBS, Meilunbio, China), and 100 units mL^−1^ penicillin and streptomycin (Beyotime Biotechnology, China). The third generation of fibroblasts was placed in serum‐free medium for 30 min and subsequently transfected with Adv‐mCherry‐GFP‐FAM134B (MOI = 10), Adv‐Flag (MOI = 50), or Adv‐Flag‐AMFR (MOI = 50) for 6 h. After transfection, the medium containing the adenovirus was replaced with DMEM supplemented with 10% FBS for 24 or 48 h. To inhibit protein synthesis, NRCFs were treated with cycloheximide (CHX, 100 µg mL^−1^) for the indicated time. Chloroquine (CQ, 10 µM) was used to inhibit lysosomes from degrading the contents of autophagosomes. and proteasome inhibitor MG132(10 µM) was used to inhibit proteasome activity.

Human embryonic kidney 293T (HEK293T) cell line was used in co‐immunoprecipitation assays to detect protein interaction between UB and FAM134B.

### siRNA Transfection

The siRNA targeting FAM134B (Sense Strand: 5′‐GCUCUUUGUCCUAAGAUUATT‐3′, Antisense Strand: 5′‐UAAUCUUAGGACAAAGAGCTT‐3′), siRNA targeting AMFR (Sense Strand: 5′‐GGCAACAUCUGGUUAUCUATT‐3′, Antisense Strand: 5′‐UAGAUAACCAGAUGUUGCCTT‐3′), and a control siRNA (Sense Strand: 5′‐ UUCUCCGAACGUGUCACGUTT‐3′, Antisense Strand: 5′‐ACGUGACACGUUCGGAGAATT‐3′) were obtained from OBiO (China). siRNAs were transfected into cells using RNAi Reagent (CALNP, China).

### Single‐Cell RNA‐seq

The heart tissues were conserved in the GEXSCOPE Tissue Preservation Solution (Singleron) and shipped to the Singleron lab with an ice pack. The specimens were washed 3 times with Hanks Balanced Salt Solution (HBSS, Gibco, Cat. No.14025‐076) and shredded into 1–2 mm pieces. Then the tissue debris was submitted to digestion with 2 ml GEXSCOPE Tissue Dissociation Solution (Singleron) at 37 °C for 15 min in a 15 ml centrifuge tube (Falcon, Cat. No.352095)with sustained agitation. Cells were filtered through 40‐micron sterile strainers(Falcon, Cat. No.352340) and centrifuged (eppendorf, 5810R) at 300 g for 5 min. Then the supernatant was removed, and the pellets were resuspended in 1 ml PBS (Hyclone, Cat. No.SA30256.01). To remove the red blood cells, which were frequently a significant portion of the cells produced, 2 mL RBC lysis buffer (Roche, Cat. No. 11 814 389 001) was added to the cell suspension according to the manufacturer's protocol. Centrifuge the cells at 500 × g for 5 min in a microfuge at 15–25 °C and resuspend in PBS (Hyclone, Cat. No.SA30256.01). The sample from the cell mixture was stained with trypan blue (Bio‐RAD, Cat. No.#1450013) and microscopically(Nikon, ECLIPSE Ts2) cell count was performed to make the concentration 1 × 105 cells mL^−1^, and once the cell viability exceeded 80%, subsequent sample processing could be performed.

Single‐cell suspensions were then loaded onto microfluidic devices, and scRNA‐seq libraries were constructed according to the Singleron GEXSCOPE protocol by GEXSCOPE Single‐Cell RNA Library Kit (Singleron Biotechnologies), which included cell lysis, mRNA trapping, labeling cells (barcode) and mRNA (UMI), reverse transcription of mRNA into cDNA, amplification, and finally fragmenting cDNA. Individual libraries were diluted to 4 nM and pooled for sequencing. Pools were sequenced on an Illumina HiSeq X with 150 bp paired end reads. Raw reads were processed with fastQC and fastp to remove low‐quality reads. Poly‐A tails and adaptor sequences were removed by cutadapt. After quality control, reads were mapped to the reference genome GRCm38 (ensembl version 99 annotation) using STAR. Gene counts and UMI counts were acquired by feature Counts software. Expression matrix files for subsequent analyses were generated based on gene counts and UMI counts.

### Co‐Immunoprecipitation

The cells were harvested and lysed following previously established protocols.^[^ ^72^ ^]^ Subsequently, the lysates were incubated overnight at 4 °C with either anti‐AMFR (#16675‐1‐AP, Proteintech, 1:100) or anti‐FAM134B (#21537‐1‐AP, Proteintech, 1:100) antibodies. Following this, 40 µl of protein G Agarose beads (Cytiva) were co‐incubated with the immune complexes for 4 h at 4 °C. After undergoing three washes with a cold wash buffer and one wash with lysis buffer, the immunoprecipitations were resuspended in 40 µl of lysis buffer and subjected to Western blot analysis to examine the indicated target proteins.

### Western Blot

Protein extraction was conducted from mouse heart tissues or cultured cells, respectively, followed by immunoblots as previously outlined. In brief, Western blot analyses employed commercially available antibodies: anti‐AMFR ((#16675‐1‐AP, Proteintech, 1:1000), anti‐FAM134B (#21537‐1‐AP, Proteintech, 1:1000), anti‐CCPG1 (#500 520, Huabio, 1:1000), anti‐SEC62(#A18589, Abclonal, 1:1000), anti‐α‐Tubulin (#HRP‐66031, Proteintech, 1:5000), anti‐GAPDH ((#HRP‐60004, Proteintech, 1:10 000), anti‐α‐SMA (# A17910, Abclonal, 1:1000), anti‐COL1A1 (#72 026, Cell Signaling Technology, 1:1000), anti‐COL3A1 (#A0817, Abclonal, 1:1000), anti‐CALNEXIN (#A24433, Abclonal, 1:1000), anti‐LC3 (#A11280, Abclonal, 1:1000), anti‐UB (#AF1705, Beyotime, 1:1000), anti‐FlAG (#AE005, Abclonal, 1:2000), anti‐MYC (#AE070, Abclonal, 1:10 000), anti‐p‐mTOR (#AP‐0115, Abclonal, 1:1000), anti‐mTOR (#A2445, Abclonal, 1:2000), p‐S6K (#AP0502, Abclonal, 1:1000), S6K (#A2190, Abclonal, 1:1000), p‐4EBP1 (#AF5806, Beyotime, 1:1000), and 4EBP1 (#AG1824, Beyotime, 1:1000), followed by incubation with peroxidase‐conjugated secondary antibodies. Signal detection utilized the enhanced chemiluminescent (ECL) system (Tanon, China), and densitometry scanning was performed using Image J software. α‐Tubulin and GAPDH were used as the loading control. Results from each experimental group were expressed as relative integrated intensity compared to the control group measured concurrently.

### Real‐Time PCR

Total RNA was isolated from the heart tissues or cultured NRCFs using an RNA isolation kit (#R701, Vazyme, China), and then converted using HiScript II Q RT SuperMix (#R223, Vazyme, China). Quantitative reverse transcription polymerase chain reaction (RT‐qPCR) reactions were performed using AceQ Universal SYBR qPCR Master Mix (#Q511, Vazyme, China) via LightCycler 96 Instrument (Roche, USA). HPRT was used as the internal reference for each sample. The primer sequences are shown in Table S3 (Supporting Information).

### Cardiac Fibroblasts Proliferation Assay

The proliferation of NRCFs was assessed through 5‐ethynyl‐2‐deoxyuridine (EdU) incorporation (Servicebio, G1602), following established procedures. The proliferative rate of NRCFs was quantified by normalizing the number of EdU‐positive cells to the total DAPI‐stained cells.

### Wound Healing Assay

For the wound healing assay, the cardiac fibroblasts transfected with Adv‐Flag or Adv‐AMFR were plated in 6‐well plates. Subsequently, the confluent monolayer cells were subjected to a linear wound area using a 200 µL pipette tip in order to assess the migratory capacity of cardiac fibroblasts. The scratched plate was then gently washed to remove detached cells, followed by treatment with TGF‐β1 or not for 24 h. Images of cells were captured at the time of injury and then again 24 h post‐injury. The migratory capacity of fibroblasts was assessed by quantifying the wound healing area using ImageJ software.

### Preparation of Cell Samples for Transmission Electron Microscopy

NRCFs were treated with TGF‐β1 and adenovirus‐mediated AMFR overexpression. Following treatment, cells were harvested from the culture dish into a centrifuge tube and centrifuged using standard cell passage procedures. The cells were then resuspended in PBS, centrifuged again, and fixed overnight at 4 °C with 2.5% glutaraldehyde. After discarding the glutaraldehyde solution, the cells were washed with PBS and fixed for 1–1.5 h at 4 °C with 1% post‐fixative. Subsequent PBS washes were performed to remove the fixative. Dehydration was carried out sequentially with 50%, 70%, and 90% ethanol, followed by a 1:1 mixture of 90% ethanol and 90% acetone, and finally 90% acetone. The cells were then treated three times with 100% acetone at room temperature for 8 min each. Resin infiltration was performed at room temperature with a 1:1 mixture of acetone and resin for 1 h, followed by a 1:2 mixture for 1 h, and finally a 1:3 mixture overnight. The resin was replaced after 3 h, and this step was repeated once. Polymerization was carried out in a 60 °C oven for 48 h. Ultrathin sections were prepared from relatively typical areas of cell ultrastructure using an ultramicrotome and stained with sodium osmium.

### Immunofluorescence

The heart tissue chips underwent dewaxing and rehydration, while cells were fixed using 4% paraformaldehyde. Following these steps, the washed heart tissue chips or cells were permeabilized with 0.1% Triton X‐100 and then blocked with 5% normal goat serum (Servicebio, China). Subsequently, they were stained with anti‐Vimentin (#5741, Cell Signaling Technology, 1:200), anti‐cTnT (#ab8295, Abcam, 1:200), anti‐CD31(#ab281583, Abcam, 1:20), anti‐CD68 (#A6554, Abclonal, 1:100), anti‐AMFR ((#16675‐1‐AP, Proteintech, 1:200), anti‐FAM134B (#HA721752, Huabio, 1:100), anti‐α‐SMA (# A17910, Abclonal, 1:100), anti‐LC3 (#ET1701‐65, Huabio, 1:500), as per the instructions. Afterward, they were incubated with Alexa Fluor 488, 594, or 647‐conjugated secondary antibodies (1:1000) (Invitrogen, USA) at room temperature for 60 min, followed by DAPI (Beyotime, China) staining. For imaging, five fields were randomly selected in each group and captured using a confocal microscope (NIKON, Japan).

### Histological Examination

Mouse hearts were collected and then immersion‐fixed in 4% buffered paraformaldehyde. Subsequently, the heart tissues were embedded in paraffin and cut into 5 µm sections. In brief, the heart tissue chips were counterstained with hematoxylin and eosin (H&E) and incubated with the Masson's Trichrome Stain Kit (Solarbio, China).

### Statistics

The data are presented as means ± SD. Statistical analyses were performed using GraphPad Prism 9.5.1. The normality of the data was estimated using the Shapiro–Wilk test. For comparisons between two groups, an unpaired *t*‐test was conducted. When comparing more than two groups, one or two‐way analysis of variance (ANOVA) was employed, followed by the post hoc Tukey's test, when the assumptions of normal distribution were met. Otherwise, the nonparametric Kruskal–Wallis test was employed for further analysis. In all cases, *p*< 0.05 was considered statistically significant.

### Ethics Approval and Consent to Participate

All animal experiments were approved by the Ethics Committee of Shanghai Jiao Tong University Affiliated Sixth People's Hospital, in accordance with the Guide for the Care and Use of Laboratory Animals established by the US National Institutes of Health (NIH Publication, 8th Edition, 2011).

## Conflict of Interest

The authors declare that they have no competing interests.

## Author Contributions

Z.W., K.N., and W.L. contributed equally to this work. X.J. was involved in the conceptualization of the study. X.J. designed and implemented the methodology. Z.X.W., K.F.N., W.L., and X.Y.W. contributed to the investigations. Z.X.W., K.F.N., W.L., X.Y.W., B.S.Y., T.X.L., Y.Z.C., and Y.Y.J. performed the visualization of the data. X.J. and Y.Y.L. acquired funding for the studies. X.J. and Y.Y.L. administered the project. Y.Y.L., Y.C., B.S.Y., T.X.L., Y.Z.C., and Y.Y.J. provided supervision for the study. X.J., Y.Y.L., Y.C., Z.X.W., K.F.N., W.L., X.Y.W., B.S.Y., T.X.L., Y.Z.C., and Y.Y.J. were involved in the review and editing of the manuscript.

## Supporting information



Supporting Information

Supporting Information

## Data Availability

All data needed to support the conclusions in the study are present in the paper and/or the Supplementary Materials.
