# Supplementary material for: ER‐phagy Activation by AMFR Attenuates Cardiac Fibrosis Post‐Myocardial Infarction via mTORC1 Pathway
Source: Adv Sci (Weinh). 2025 Jul 17;12(37):e04552. doi: 10.1002/advs.202504552 (PMC12499418; doi:10.1002/advs.202504552)
Supplement: Supplementary file 1 — Supporting Information [file ADVS-12-e04552-s001.docx]

**ER-phagy Activation by AMFR Attenuates Cardiac Fibrosis Post-Myocardial Infarction via mTORC1 Pathway**

Zhixiang Wang^1^†, Kaifan Niu^1,2^†, Wei Liu^1^†, Xinyun Wang^1^†, Boshen Yang^1^, Taixi Li^1^, Yizhi Chen^1^, Yuanyuan Jin^3^, Yu Chen^1*^, Yangyi Lin^1*^, Xian Jin^1*^

^1^Department of Cardiology, Shanghai Sixth People’s Hospital Affiliated to Shanghai Jiao Tong University School of Medicine, Shanghai 200233, China.

^2^Tongren Hospital, Shanghai Jiao Tong University School of Medicine, Shanghai 200050, China.

^3^International Medical College of Chongqing Medical University, Chongqing 400010, China.

†These authors contributed equally to this work.

*****Corresponding authors:

Xian Jin, Ph. D.

Email: [jinxianian@sjtu.edu.cn](mailto:jinxianian@sjtu.edu.cn)

Yangyi Lin, Ph. D.

Email: 7250013351@shsmu.edu.cn

Yu Chen, Ph. D.

Email: chenyu007@sjtu.edu.cn

Table S1. Quality control of scRNA-seq dataset.

|  | **WT** | **AMFR_KO** |
| --- | --- | --- |
| Estimated Number of Cells | 5,218 | 10,622 |
| Fraction Reads in Cells | 57.98% | 59.47% |
| Mean Reads per Cell | 72,208 | 35,758 |
| Median UMI per Cell | 1,625 | 1,903 |
| Total Genes | 21,268 | 22,415 |
| Median Genes per Cell | 815 | 875 |
| Saturation | 78.77% | 72.65% |
| Fraction of cells have mito gene percent>5% | 6.46% | 8.03% |
| Fraction of cells have mito gene percent>10% | 1.8% | 1.3% |
| Fraction of cells have mito gene percent>15% | 0.82% | 0.34% |
| Fraction of cells have mito gene percent>20% | 0.57% | 0.13% |
| Fraction of cells have mito gene percent>30% | 0.27% | 0.06% |
| Fraction of cells have mito gene percent>50% | 0.04% | 0.03% |

Table S2. The genotyping strategies of Amfr.

| **Primer** | **Sequence** | **Product** |
| --- | --- | --- |
| Primer1 | F1: 5’- TCCTTTGTTCTTCTGCTTCTGGTA-3’ | 265 bp |
|  | R1: 5’- AGCCTTTCAGGACTGTACCAAT-3’ |  |
| Primer2 | F2: 5’- TCTGTGTCATTAATAAGTGGGGTG-3’ | 583 bp |
|  | R1: 5’- AGCCTTTCAGGACTGTACCAAT -3’ |  |

*Amfr*^-/-^: one band with 265 bp; *Amfr*^+/-^: two bands with 265 bp and 583 bp; *Amfr*^+/+^: one band with 583 bp

| **Primer sequence** | **Forward** | **Reverse** |
| --- | --- | --- |
| Rat Hprt | GCTGAAGATTTGGAAAAGGTGT | ACAGAGGGCCACAATGTGAT |
| Rat Acta2 | CAGTCGCCATCAGGAACCTC | TTGGCCCATTCCAACCATCA |
| Rat Col1a1 | CCGATGGATTCCAGTTCGAGT | GGGACTTCTTGAGGTTGCCA |
| Rat Col3a1 | GCCTACATGGATCAGGCCAA | CACCAGTGTGTTTAGTGCAGC |
| Mus Hprt | GTTGGATACAGGCCAGACTTTGTT | GATTCAACTTGCGCTCATCTTAGGC |
| Mus Acta2 | AGCCATCTTTCATTGGGATGG | CCCCTGACAGGACGTTGTTA |
| Mus Col1a1 | CGATGGATTCCCGTTCGAGT | CGATCTCGTTGGATCCCTGG |
| Mus Col3a1 | TGACTGTCCCACGTAAGCAC | GAGGGCCATAGCTGAACTGA |
| Mus Retreg1 | ACGTGAGAGATCTGAAGCAG | AAGTGTCTGTCTGCGGAGTG |
| Mus Atl3 | GTCCTCCGTTTCCTCTACGC | CCATTTTCCATGGCATCACCTGT |
| Mus Sec62 | GACAAAGATGGAGAGCTTAGGAA | ATGACAAGGAGATGGGGAGGA |
| Mus Ccpg1 | AGCCAGAACCCGTCTTTTCA | TGGCGTTTACTTGGCTCCTT |
| Mus Calcoco1 | CTATGACGTGGCCAGTGCTTT | CTCCTTGCAGATGGGACACT |
| Mus Tex264 | GTCCCGCCGCGAAAGG | TCCAGTACTCCGGCGCTCA |

Table S3. The primer sequences for qRT-PCR.


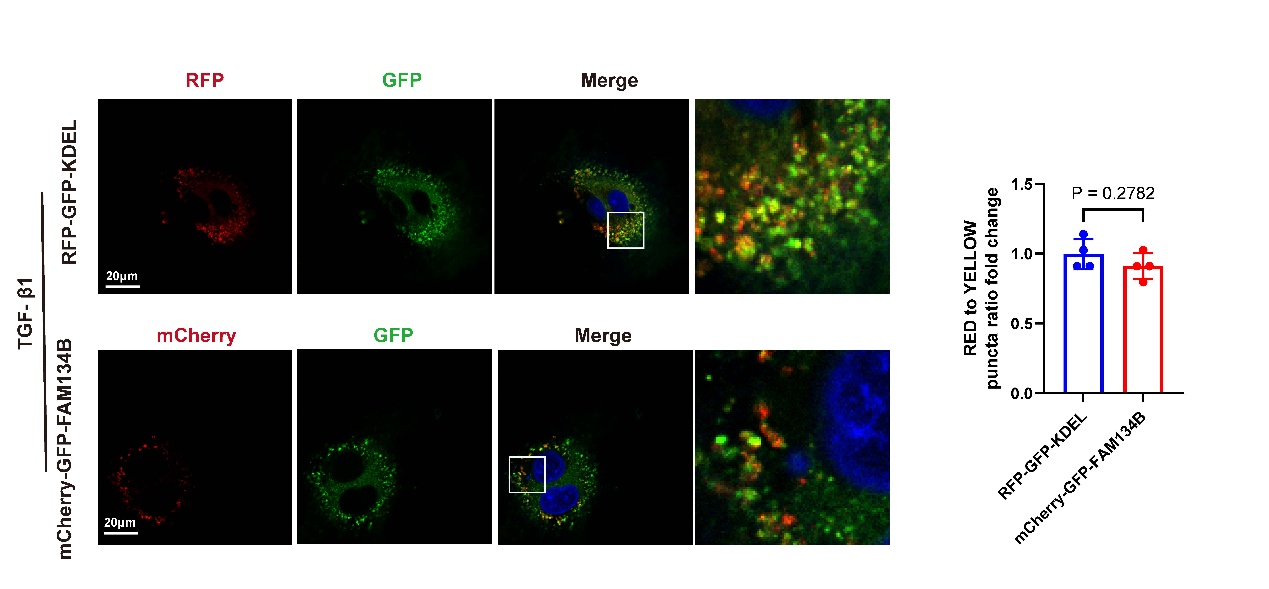


Figure S1. Adult mouse primary cardiac fibroblast (ACFs) isolated from WT mice were transfected with RFP–GFP–KDEL mCherry-GFP-FAM134B for 24h. Cells were then untreated or treated with TGF-β1(10ng/ml) for 24h. The results showed that the mCherry/mCherry-GFP puncta ratio was close to RFP/RFP-GFP puncta ratio, suggesting that ER-phagy is dependent on FAM134B in fibroblast treated with TGF-β1. Data are presented as mean ± SD. The data were analyzed using an unpaired t-test.


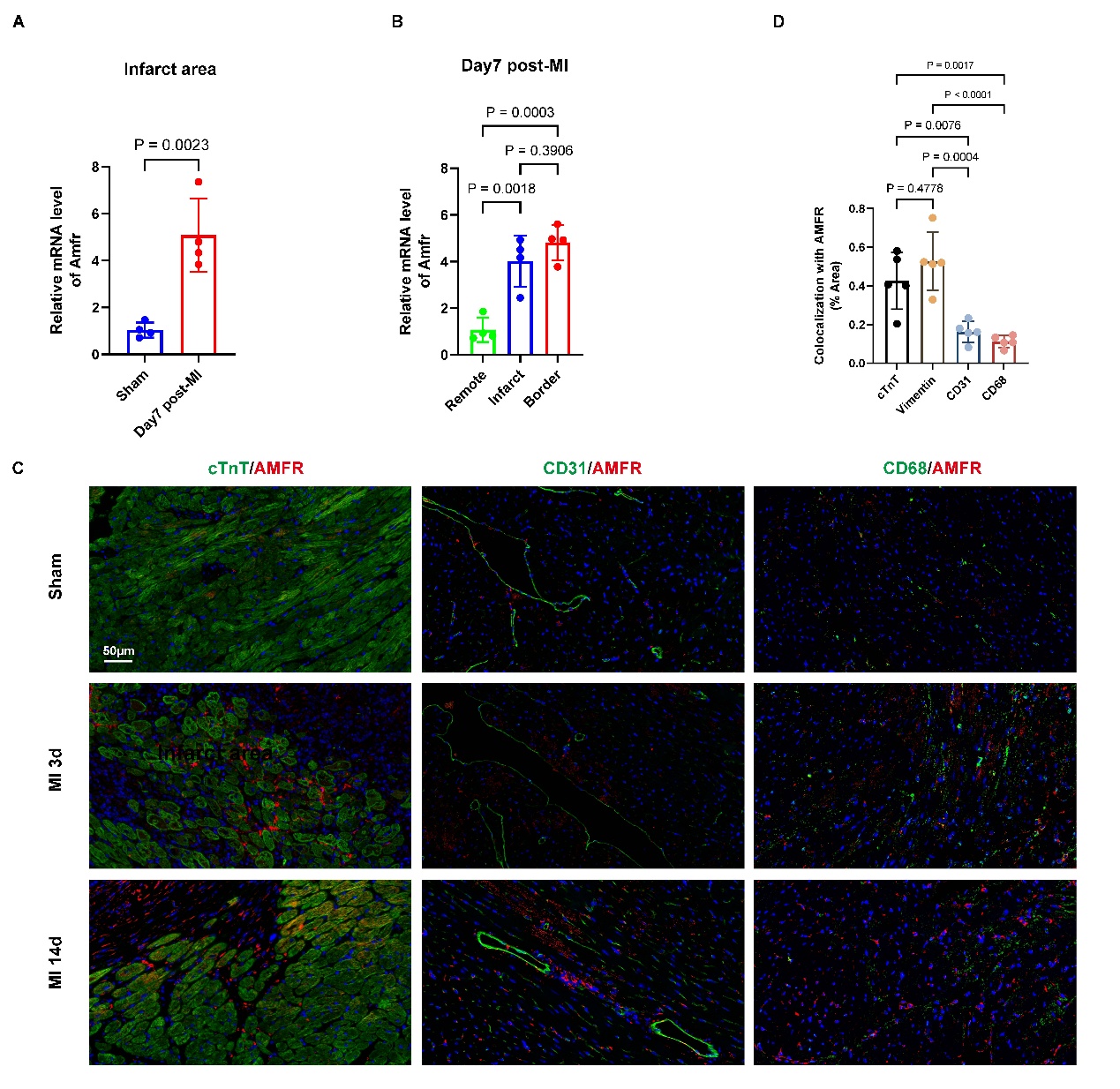


Figure S2. **A** Expression of AMFR was measured using RT‐qPCR in the infarct area of WT mice at day7 post-MI as well as in sham control. n = 4. **B** mRNA level of AMFR in the infarct area, border area and remote area of heart at day7 post myocardial infarction. n = 4. **C** Immunofluorescence co-staining for AMFR (red) with cTnt, CD31, CD68(green) and DAPI (blue) in the mouse heart tissue sections from sham mice or MI mice at 3 days, 14 days post MI. **D** Quantitative analysis of the co-localization between AMFR and cTnT, Vimentin, CD31, and CD68 in mouse heart tissue sections obtained from mice at 14 days post-myocardial infarction. n=5. Data are presented as mean ± SD. The data shown in **A** were analyzed using an unpaired t-test. The data shown in **B** and **D** was analyzed using one-way ANOVA corrected by the post hoc Turkey’s test.


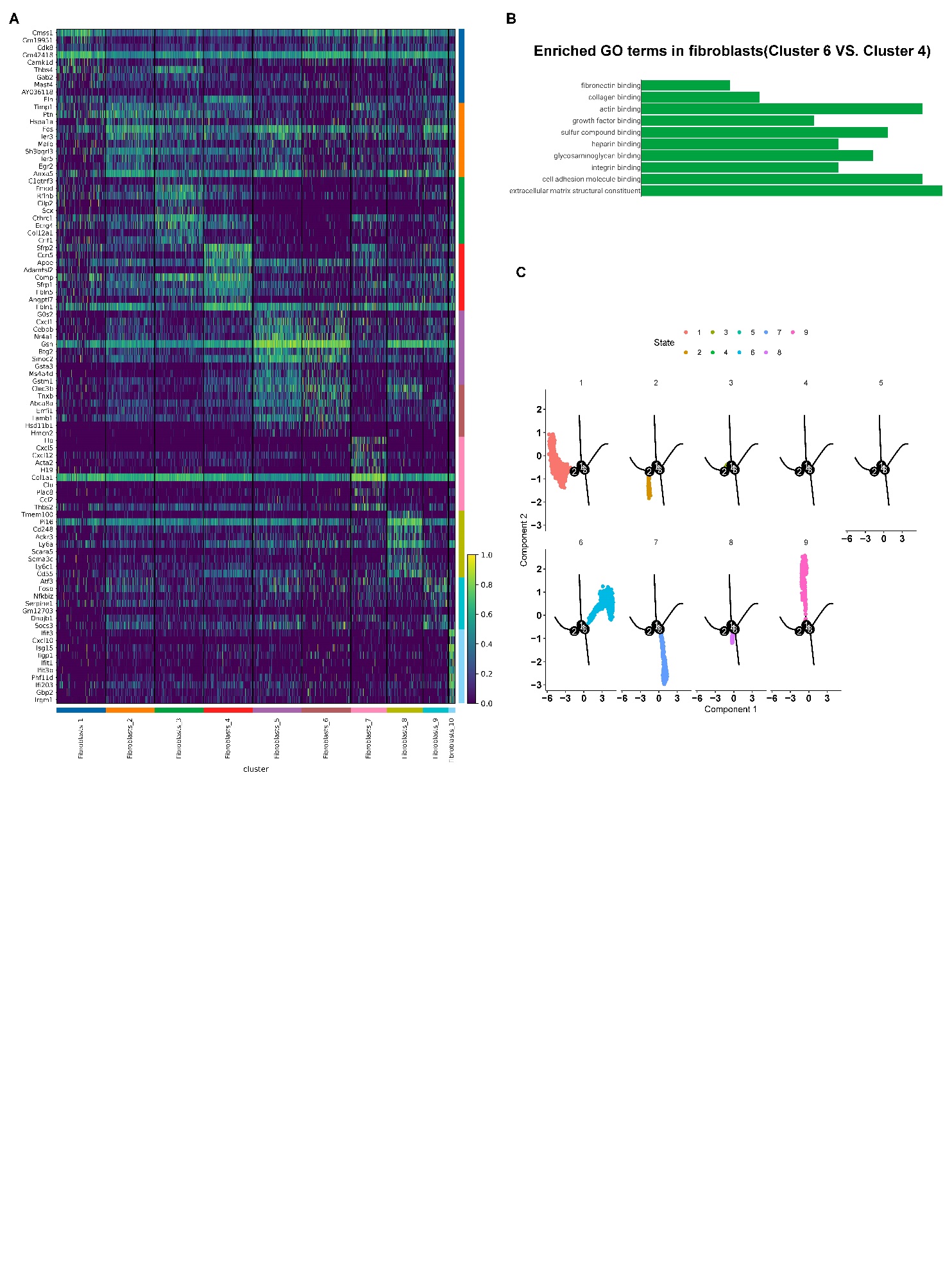


Figure S3. **A** Hierarchical clustering reveals the genes that define the identity of cluster 6 (quiescent) and cluster 4 (myofibroblast). **B** Bar plot visualization of Gene ontology biological processes that are enriched in Cluster6 vs. Cluster4. **C** The distribution of 9 cell states across pseudotime.


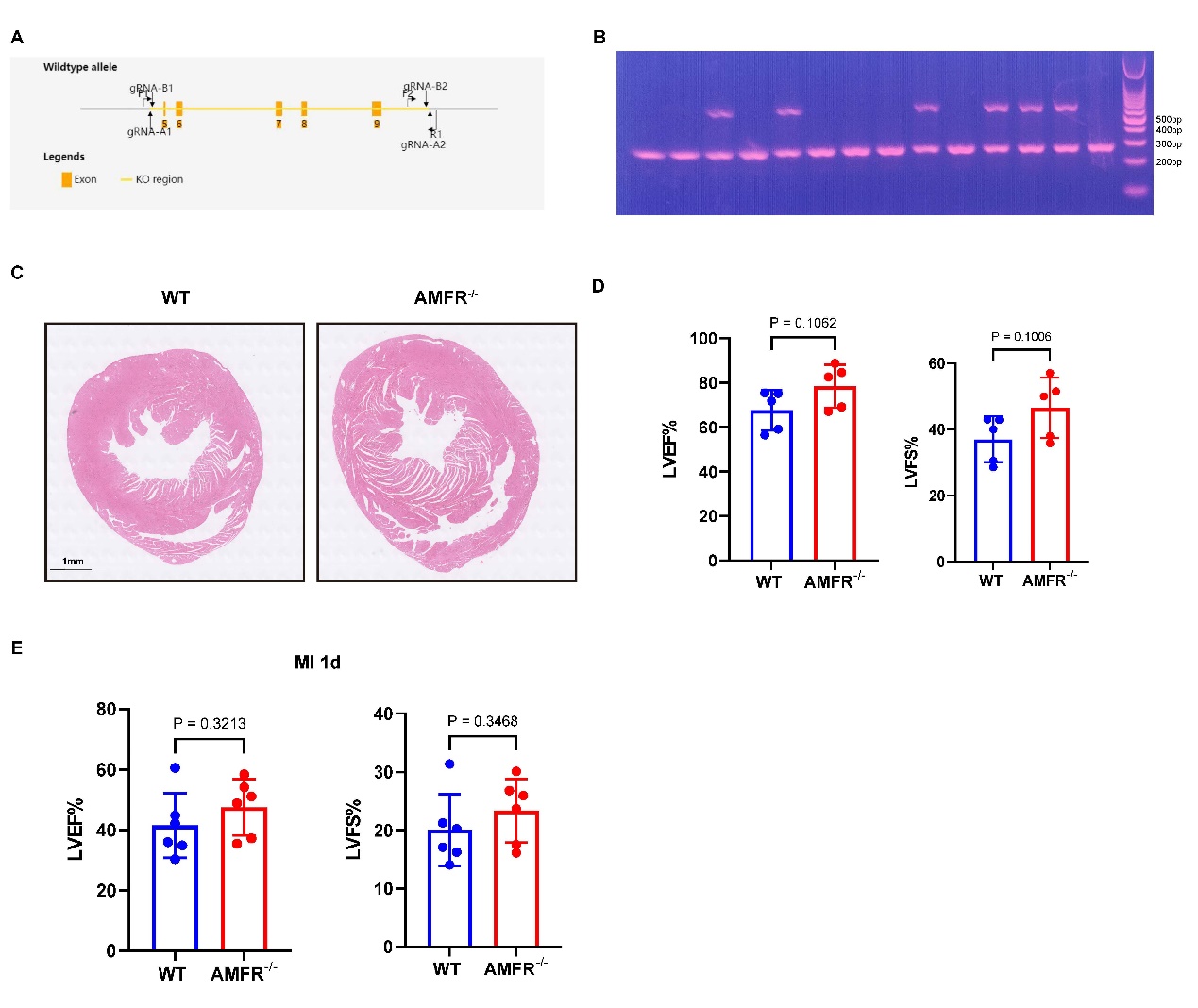


Figure S4. **A** The regimen of AMFR knockout strategy. **B** Representative image of PCR results for Amfr genotyping. The presence of a single band with 265 bp signified successful knockout of Amfr. **C** Hematoxylin-Eosin staining of heart tissues from WT mice and AMFR^-/-^ mice. **D** Ejection fraction (EF%) and fractional shortening (FS%) of WT mice and AMFR^-/-^ mice before subjecting to the surgery, n=5. **E** Ejection fraction (EF%) and fractional shortening (FS%) of WT mice and AMFR^-/-^ mice 1 day after MI surgery, n=6. Data are presented as mean ± SD. The data shown in **D-E** were analyzed using an unpaired t-test.


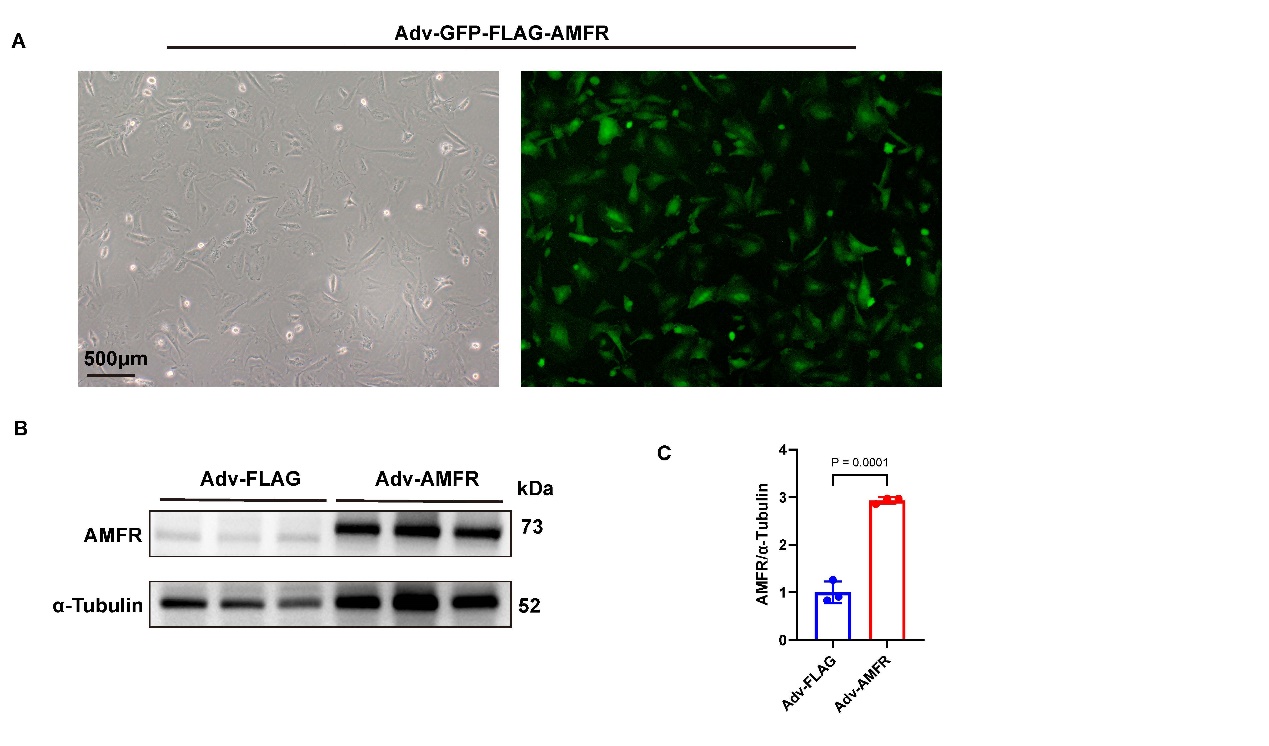


Figure S5. **A** Representative bright-field image and fluorescence image of NRCFs transfected with Adv-GFP-FLAG-AMFR for 48h. **B-C** Representative Western blots and statistical result of AMFR in NRCFs transfected with Adv-FLAG or Adv-AMFR for 48h. n=3. Data are presented as mean ± SD. The data shown in **C** were analyzed using an unpaired t-test.


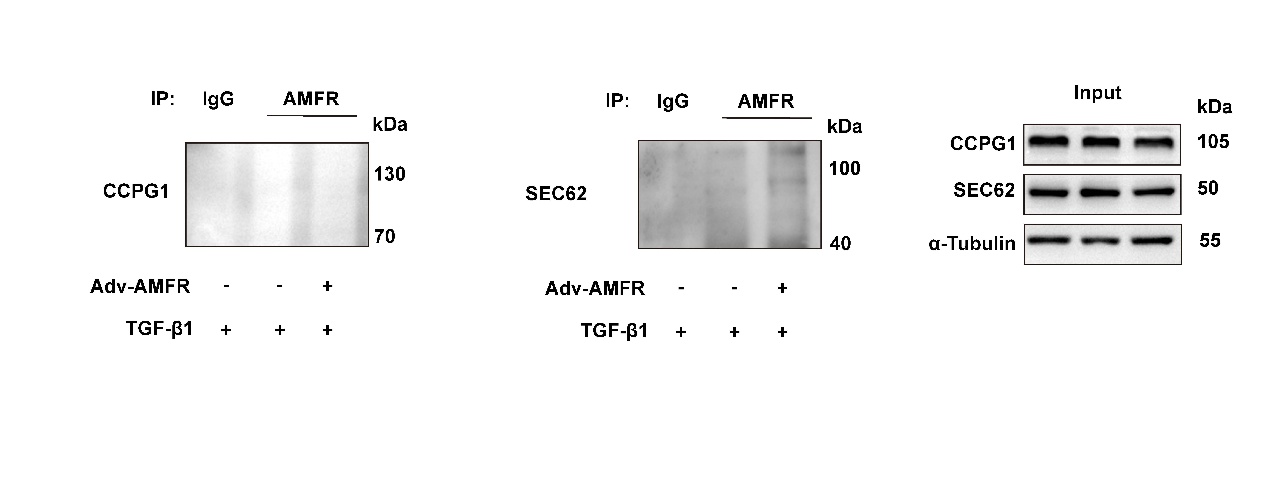


Figure S6. NRCFs were transfected with Adv-Flag or Adv-AMFR for 48 h. Immunoprecipitation with the antibody against AMFR, and followed by immunoblot with anti- CCPG1 and anti- SEC62 antibody. IgG as a negative control.


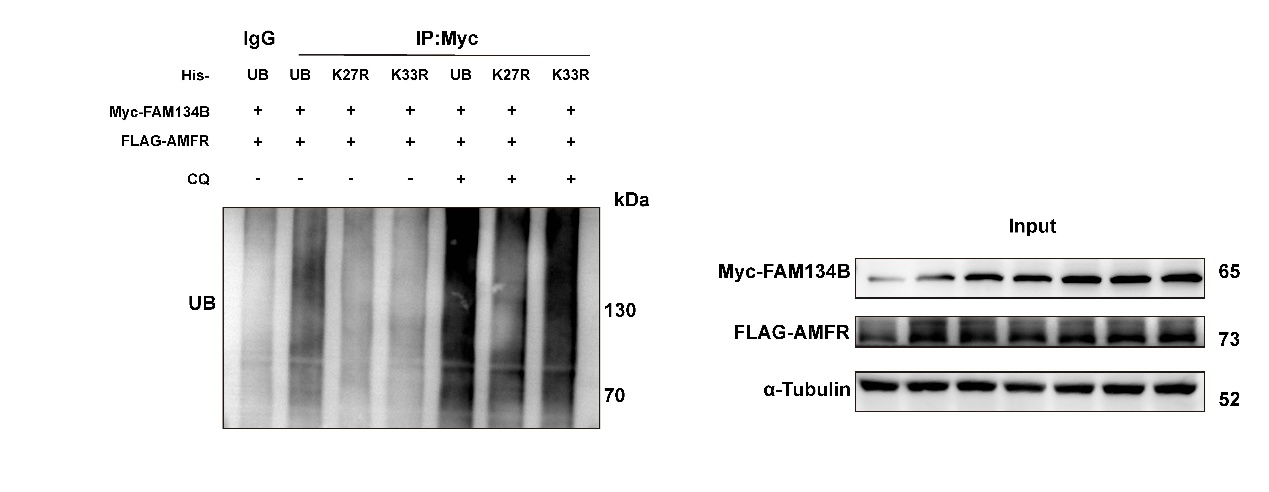


Figure S7. HEK293T cells transduced with His-Ub or His-K27R-UB, His-K33R-UB and MYC-FAM134B, Flag-AMFR vectors were treated with or without chloroquine (CQ). Cell lysates were subjected to immunoprecipitation with the antibody against MYC, and followed by immunoblot with anti-UB antibody. IgG as a negative control.


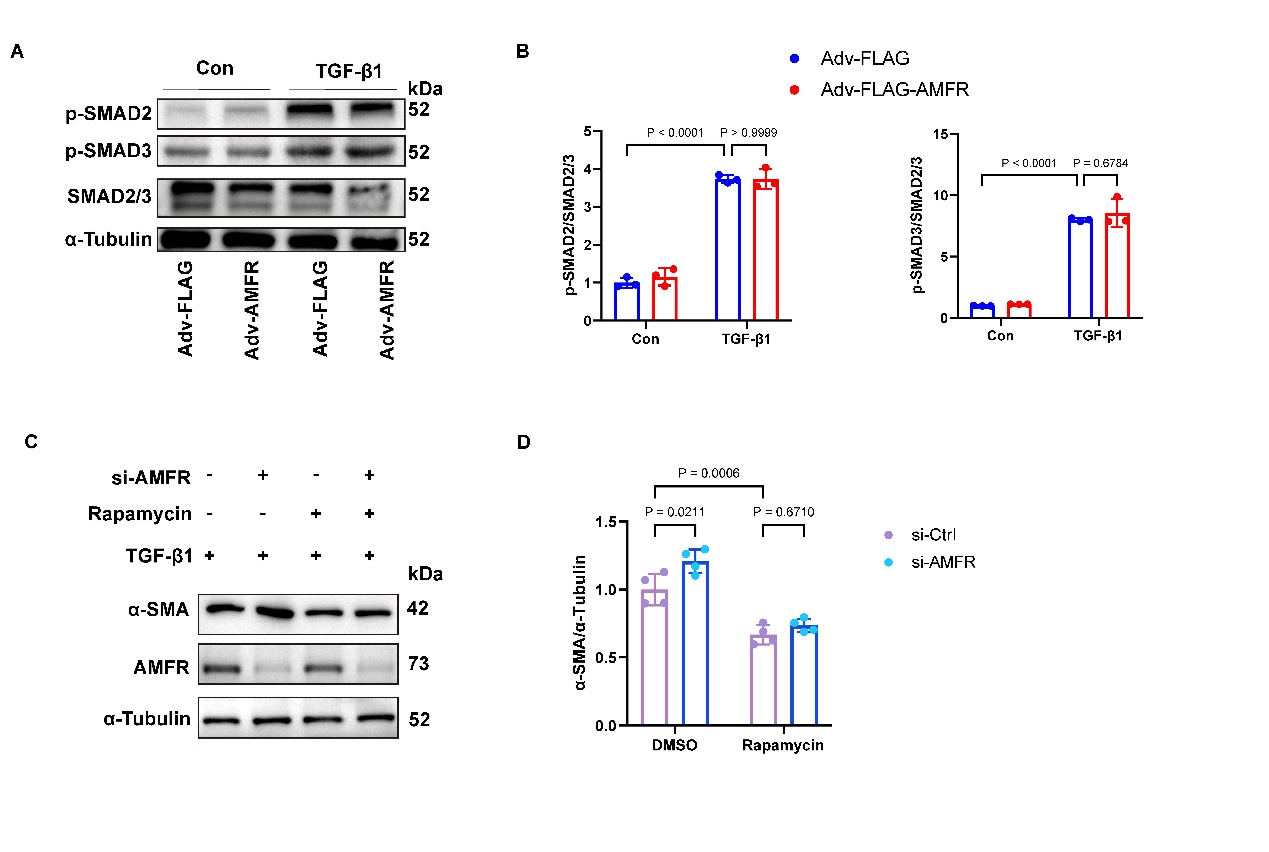


Figure S8. NRCFs were isolated from 1 to 3-day-old neonatal Sprague-Dawley rats. **A-B** NRCFs were transfected with Adv-AMFR or Adv-Flag for 48 h before being treated with TGF-β1(10 ng/mL) for 24 h. Representative Western blots and statistical results of of p-SMAD2, p-SMAD3 and SMAD2/3 in NRCFs, n = 3. **C-D** NRCFs were transfected with si-Ctrl or si-AMFR for 24 h, followed by treatment with Rapamycin (0.1 μM) for 24 h, and then exposed to TGF-β1 (10 ng/mL) for an additional 24 h. Representative Western blots and statistical results of α-SMA in NRCFs, n = 4. Data are presented as mean ± SD. The data were analyzed using two-way ANOVA corrected by the post hoc Turkey’s test.


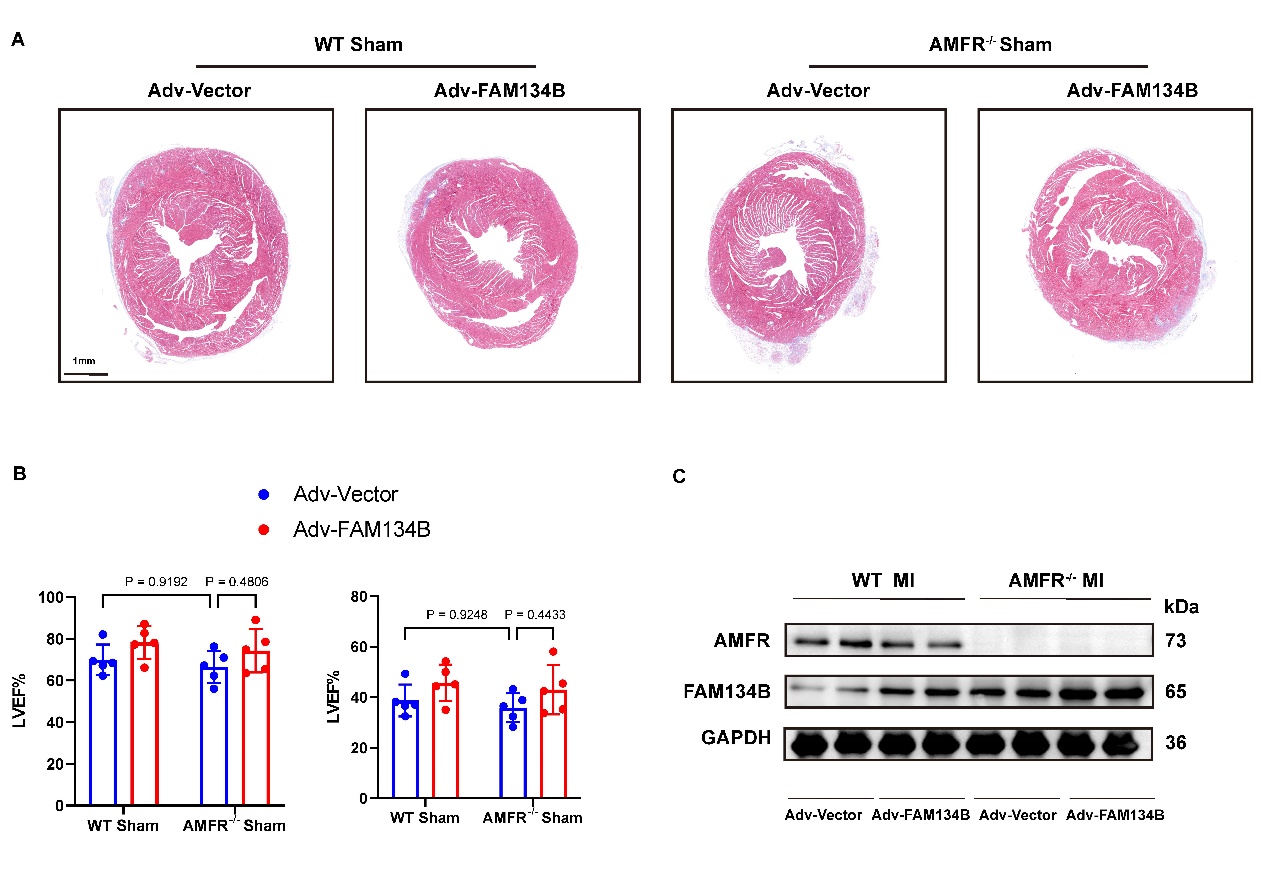


Figure S9. 8-week-old WT or AMFR-knockout male mice were transfected with Adv-FAM134B or Adv-Vector via intracardiac injection for one week before being subjected to Sham surgery or MI surgery, and heart tissues were harvested 2 weeks post-surgery. **A** Masson’s trichrome staining was performed to detect myocardial collagen deposition of WT or AMFR-knockout mice transfected with Adv-FAM134B or Adv-Vector, following Sham surgery. **B** Quantification of echocardiographic parameters of ejection fraction (EF%) and fractional shortening (FS%) of WT or AMFR-knockout mice transfected with Adv-FAM134B or Adv-Vector, following Sham surgery. n=5. **C** Representative Western blots of AMFR and FAM134B in heart tissues from WT or AMFR-knockout mice transfected with Adv-FAM134B or Adv-Vector, following MI surgery. Data are presented as mean ± SD. The data were analyzed using two-way ANOVA corrected by the post hoc Turkey’s test.
