# Supplementary figures and images for: ER‐phagy Activation by AMFR Attenuates Cardiac Fibrosis Post‐Myocardial Infarction via mTORC1 Pathway
Source: Adv Sci (Weinh). 2025 Jul 17;12(37):e04552. doi: 10.1002/advs.202504552 (PMC12499418; doi:10.1002/advs.202504552)

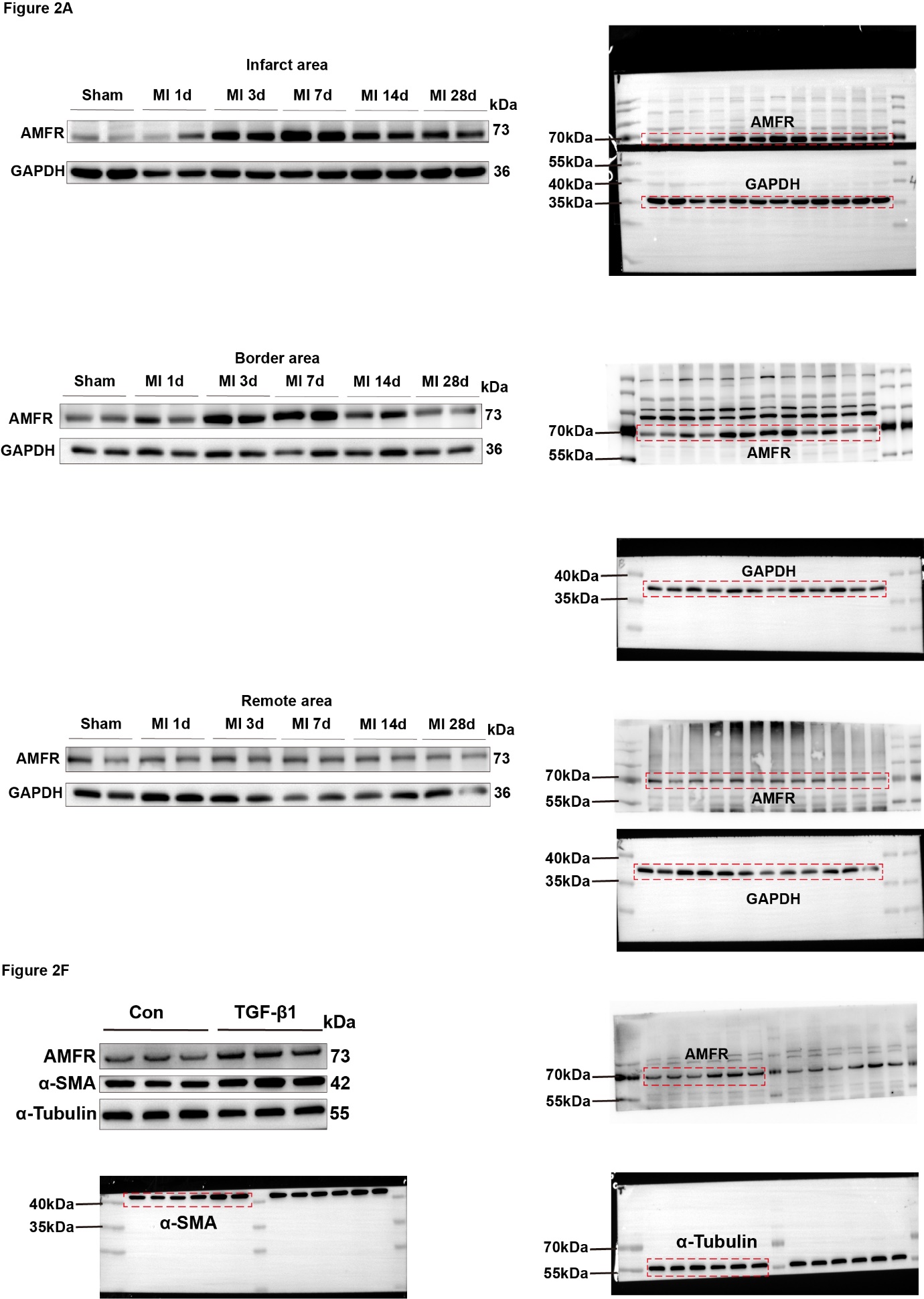


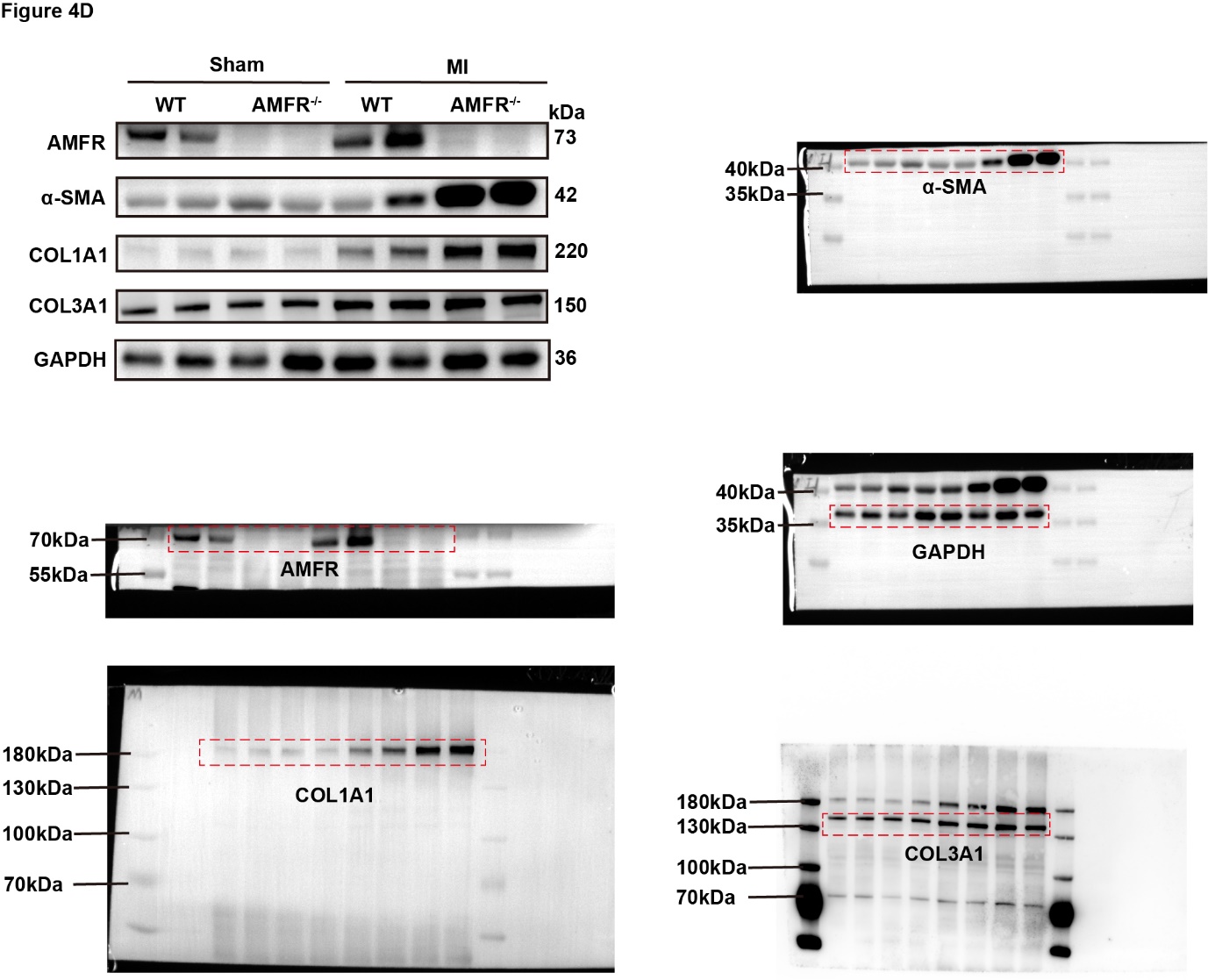


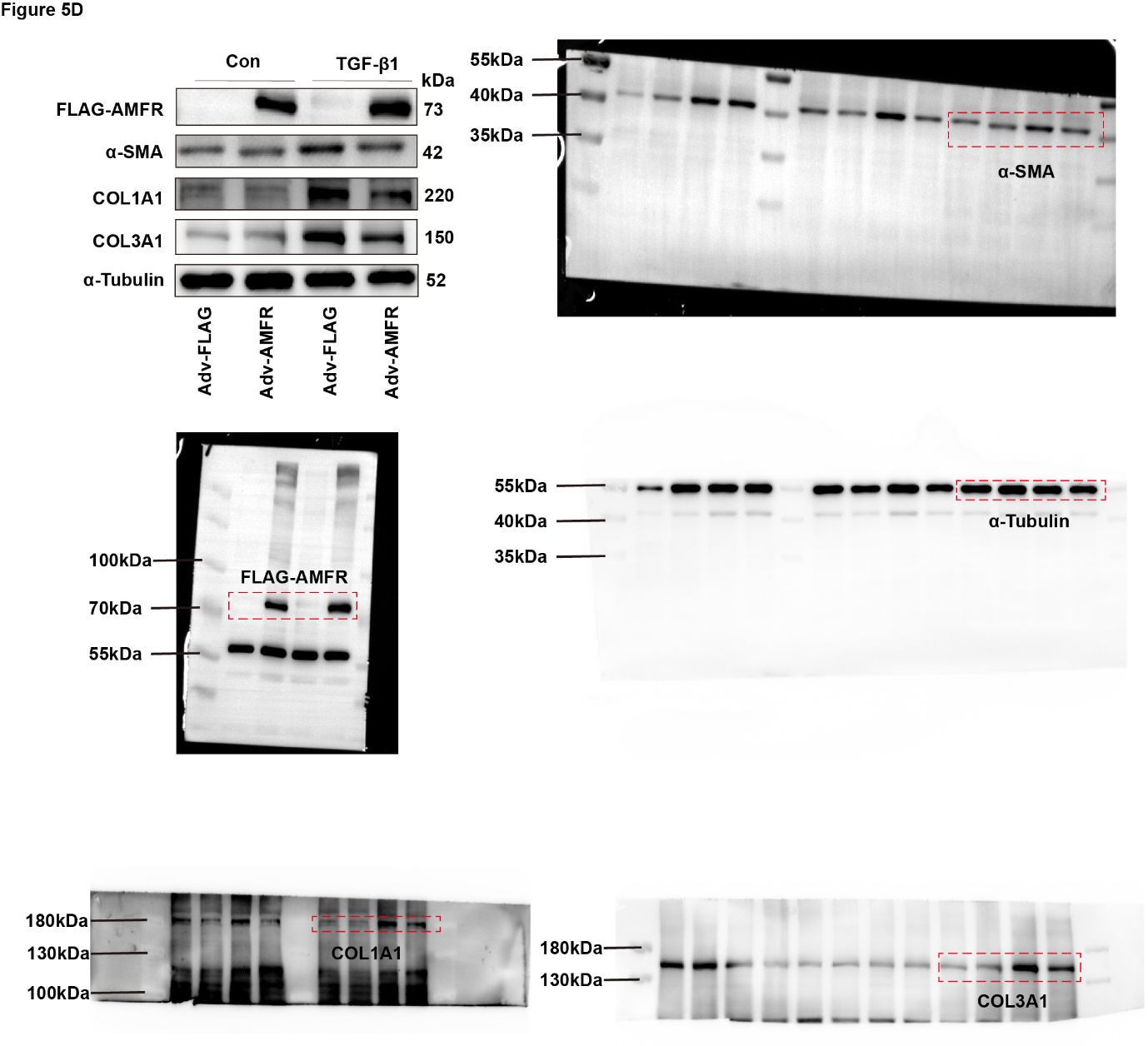


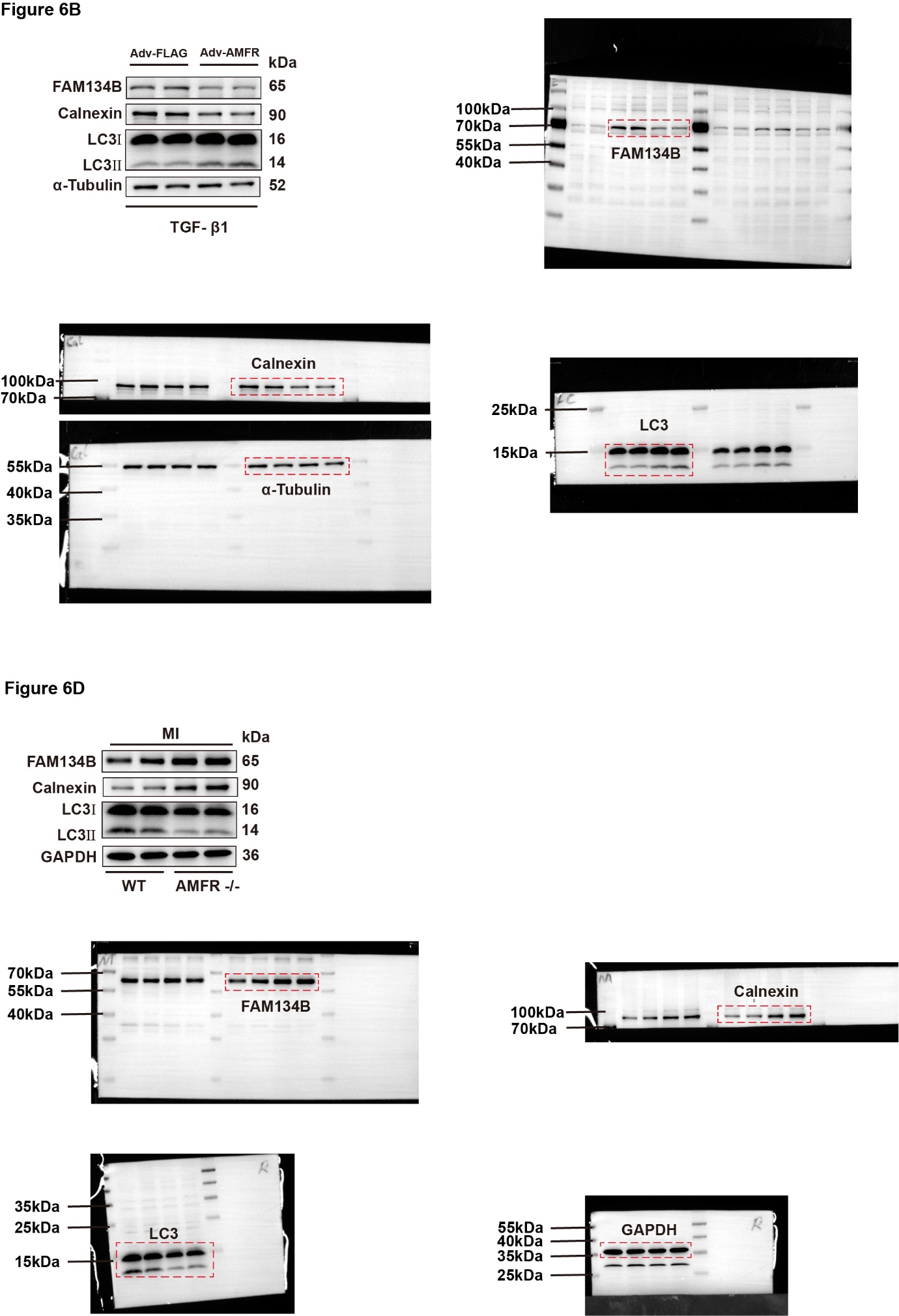


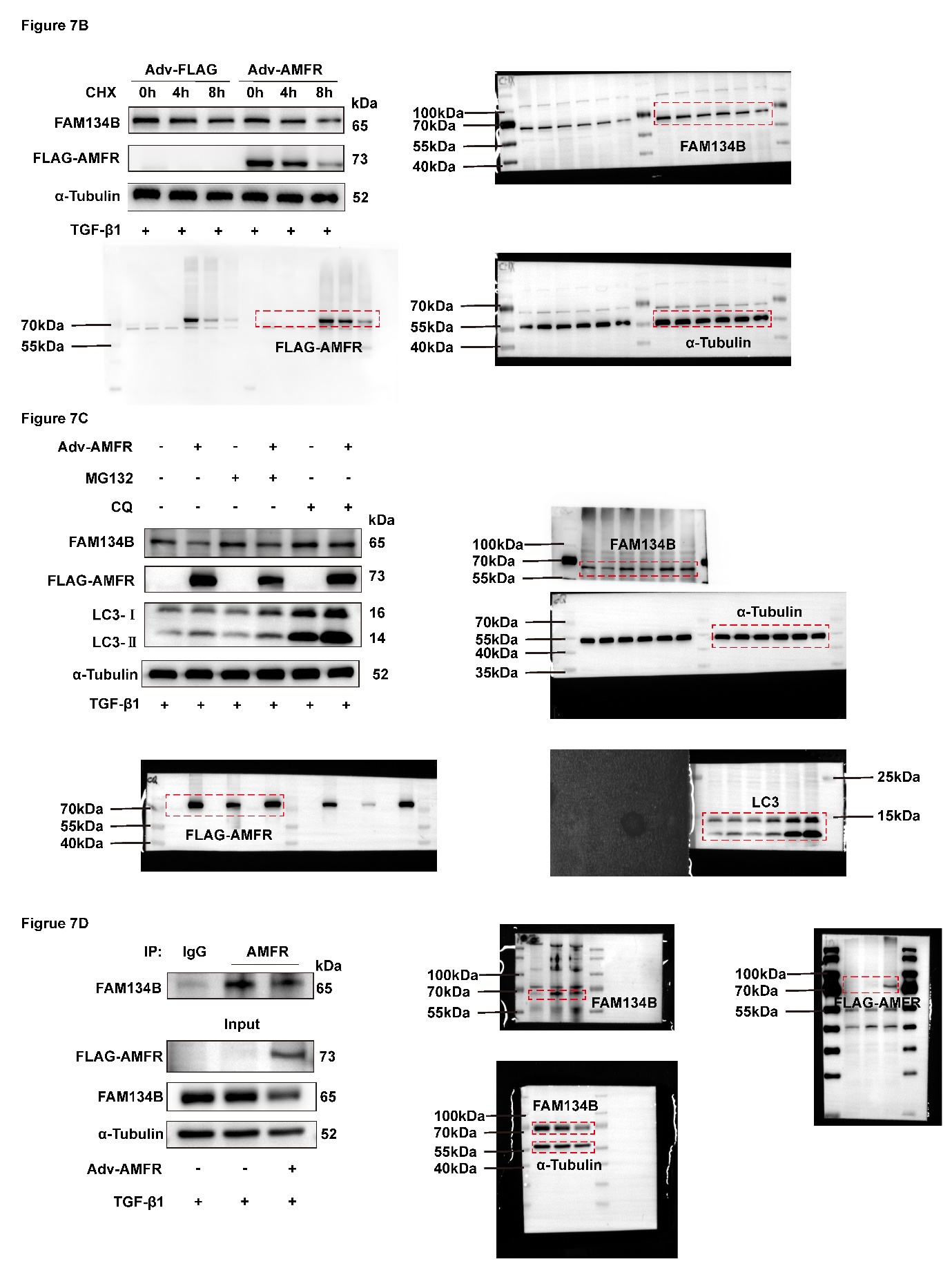


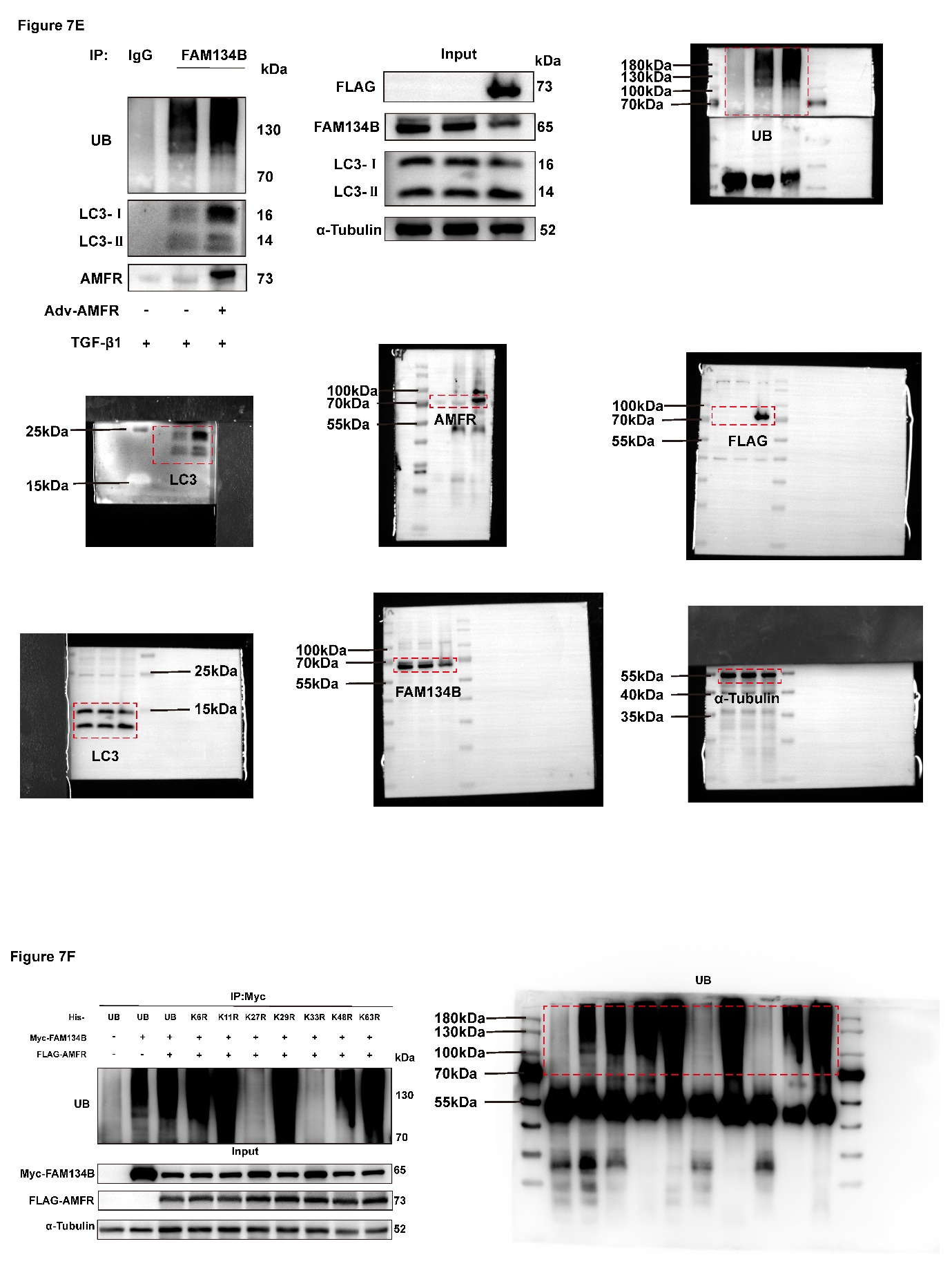


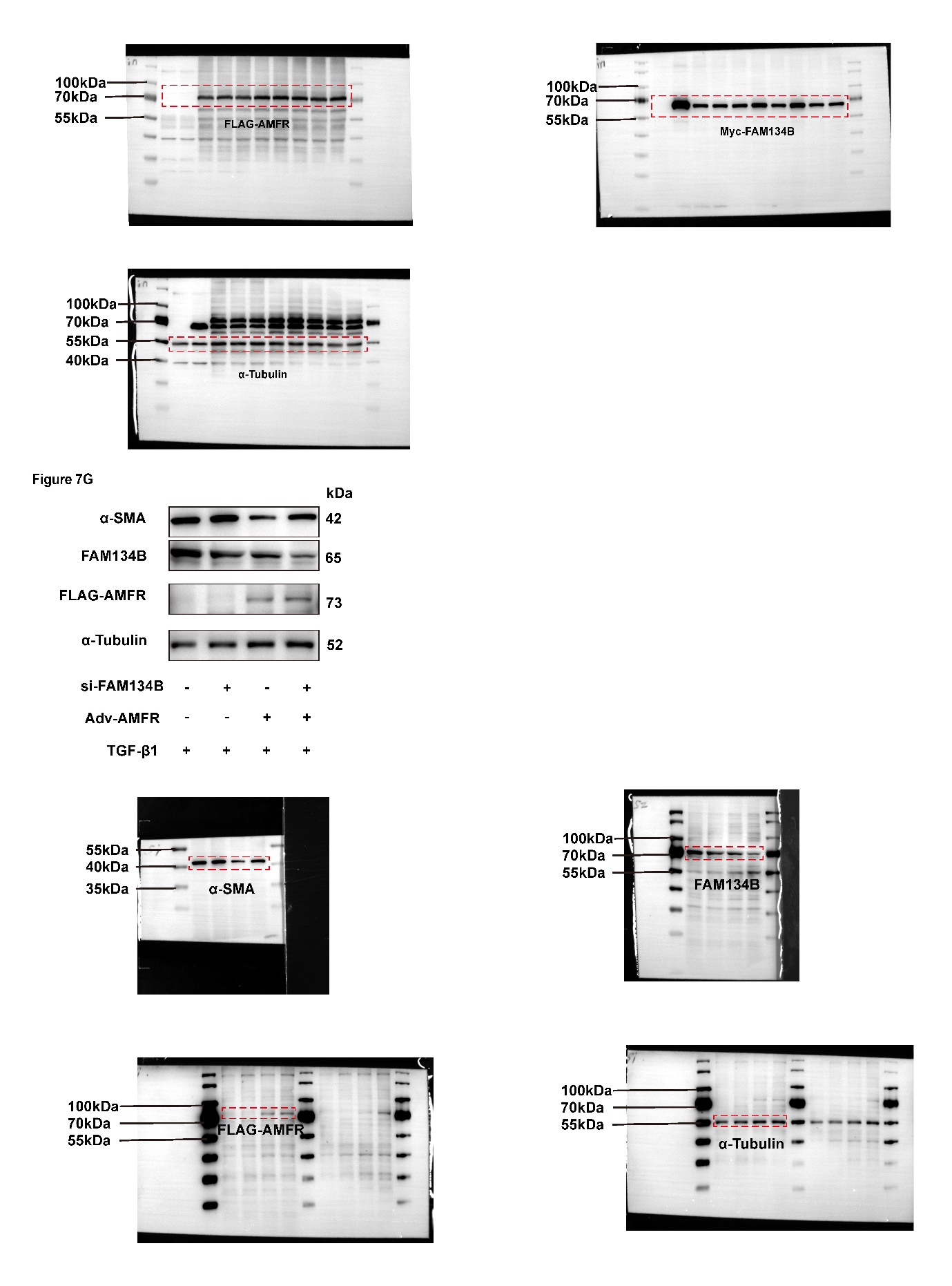


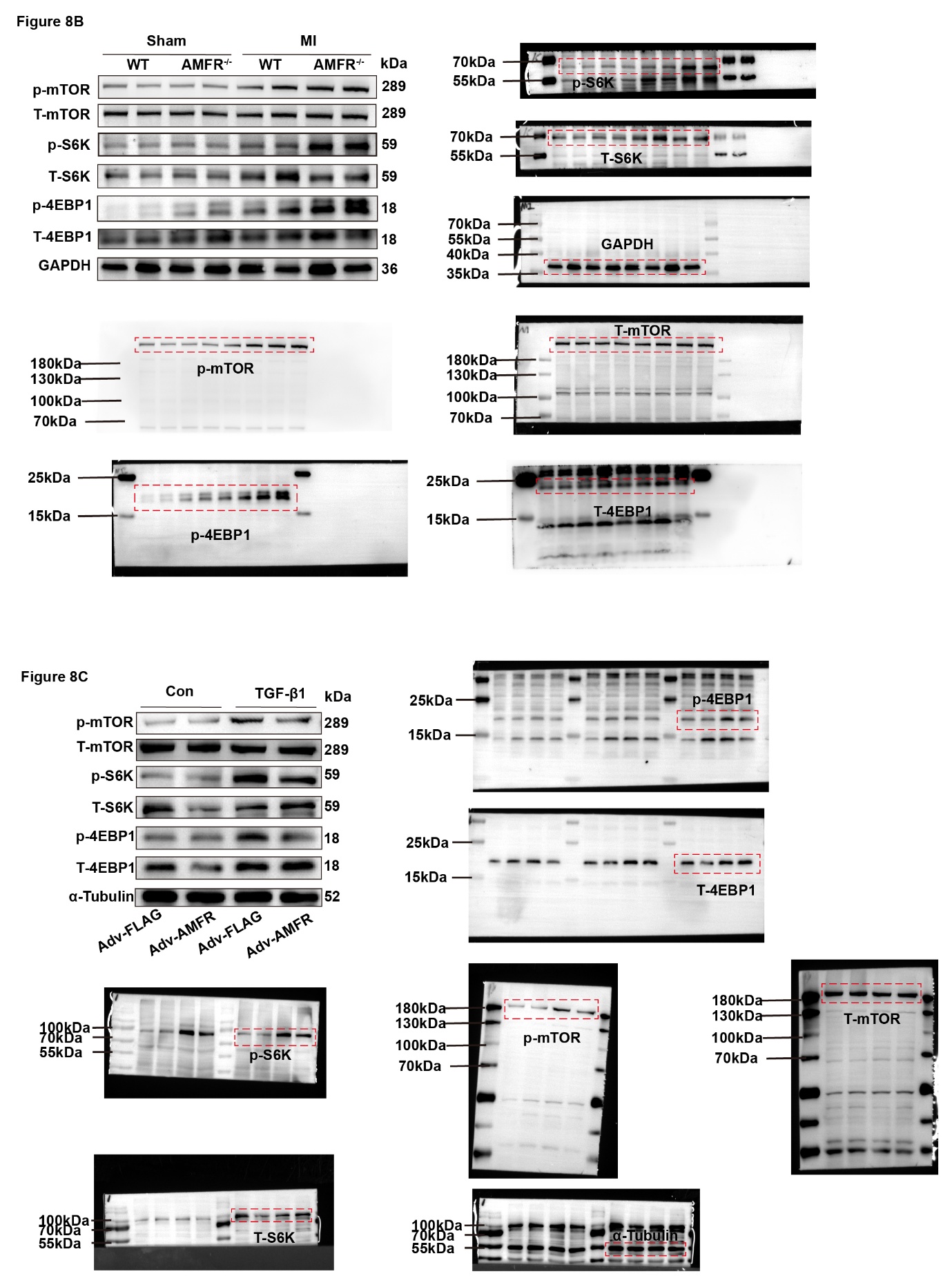


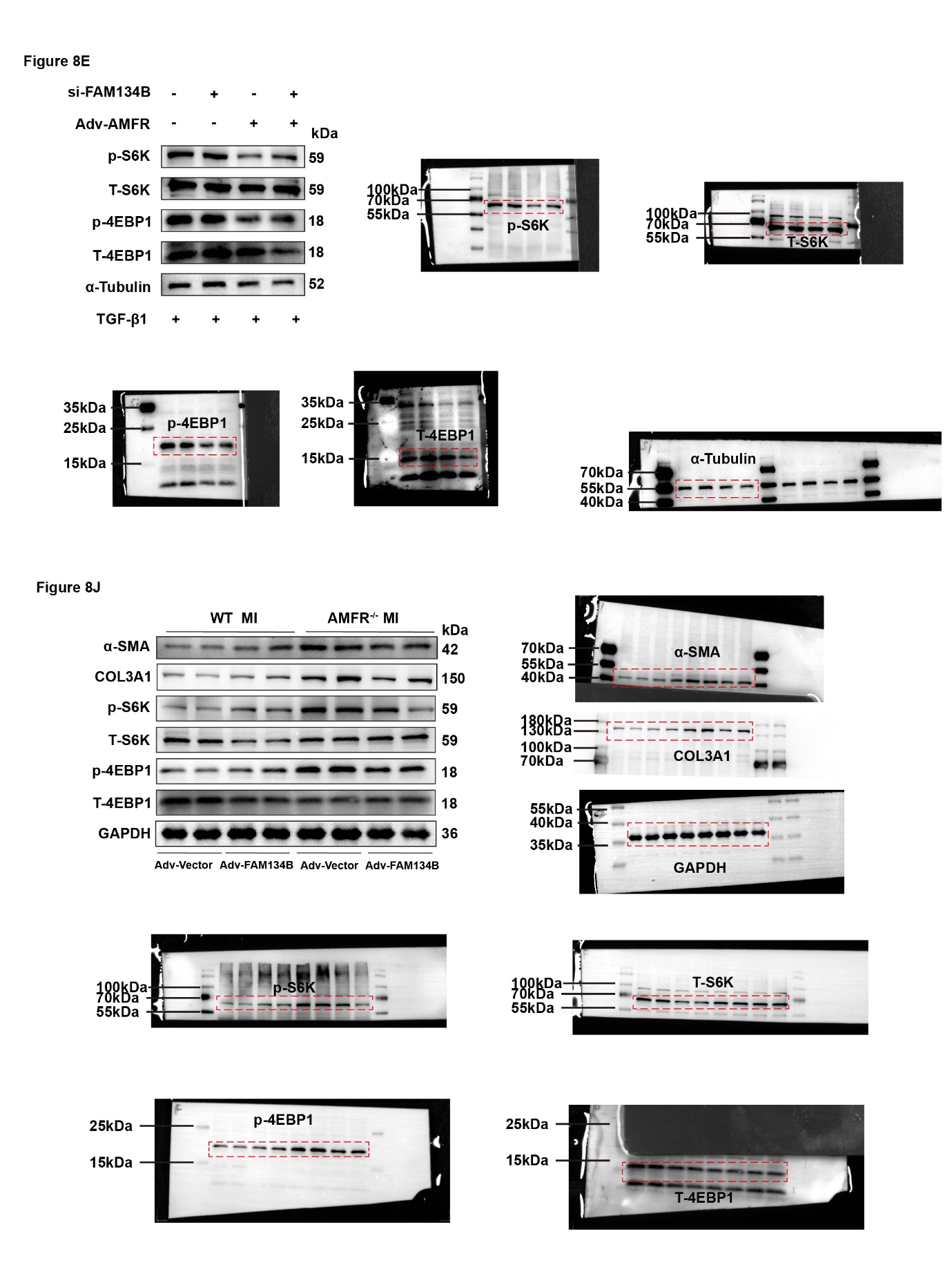


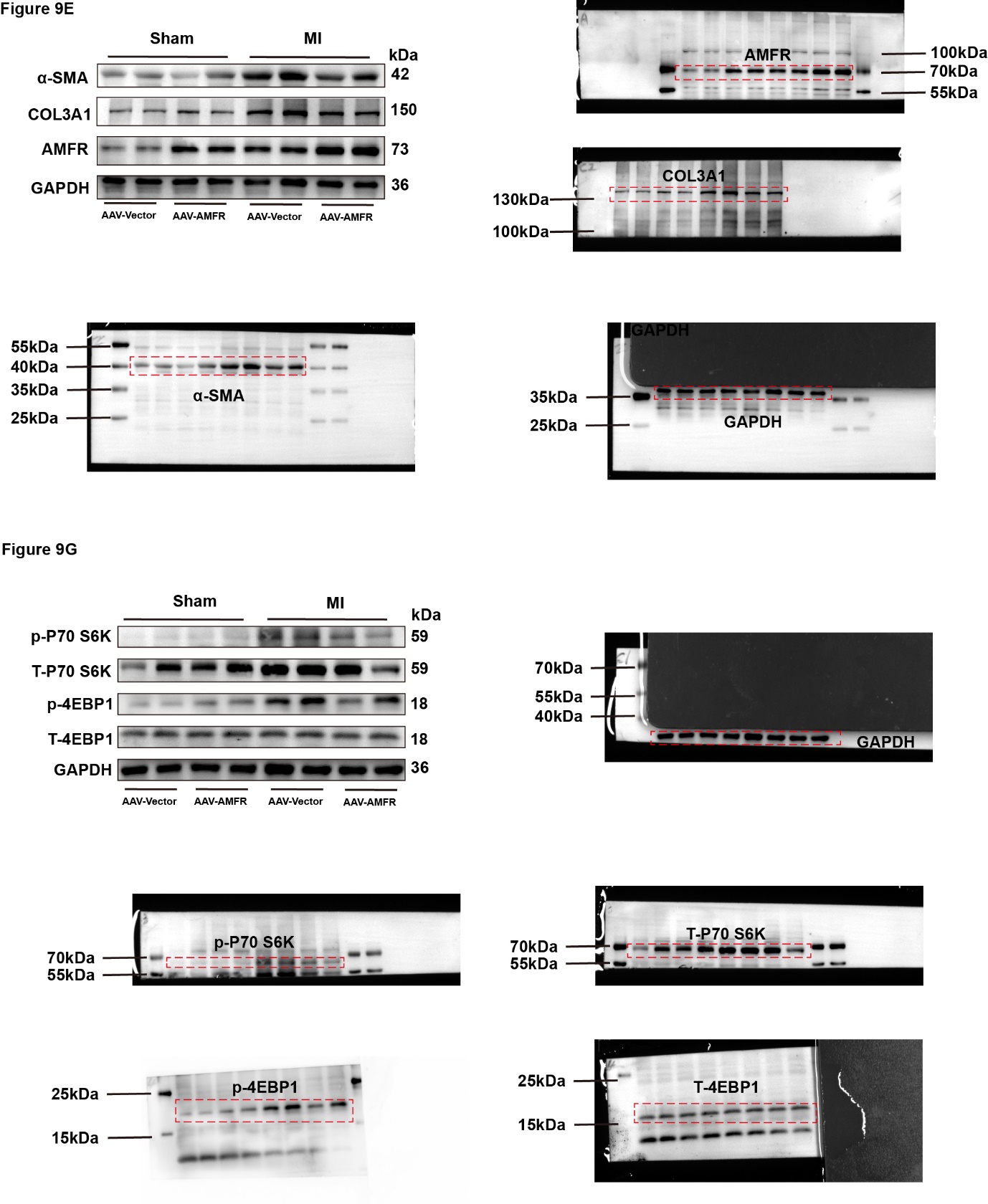


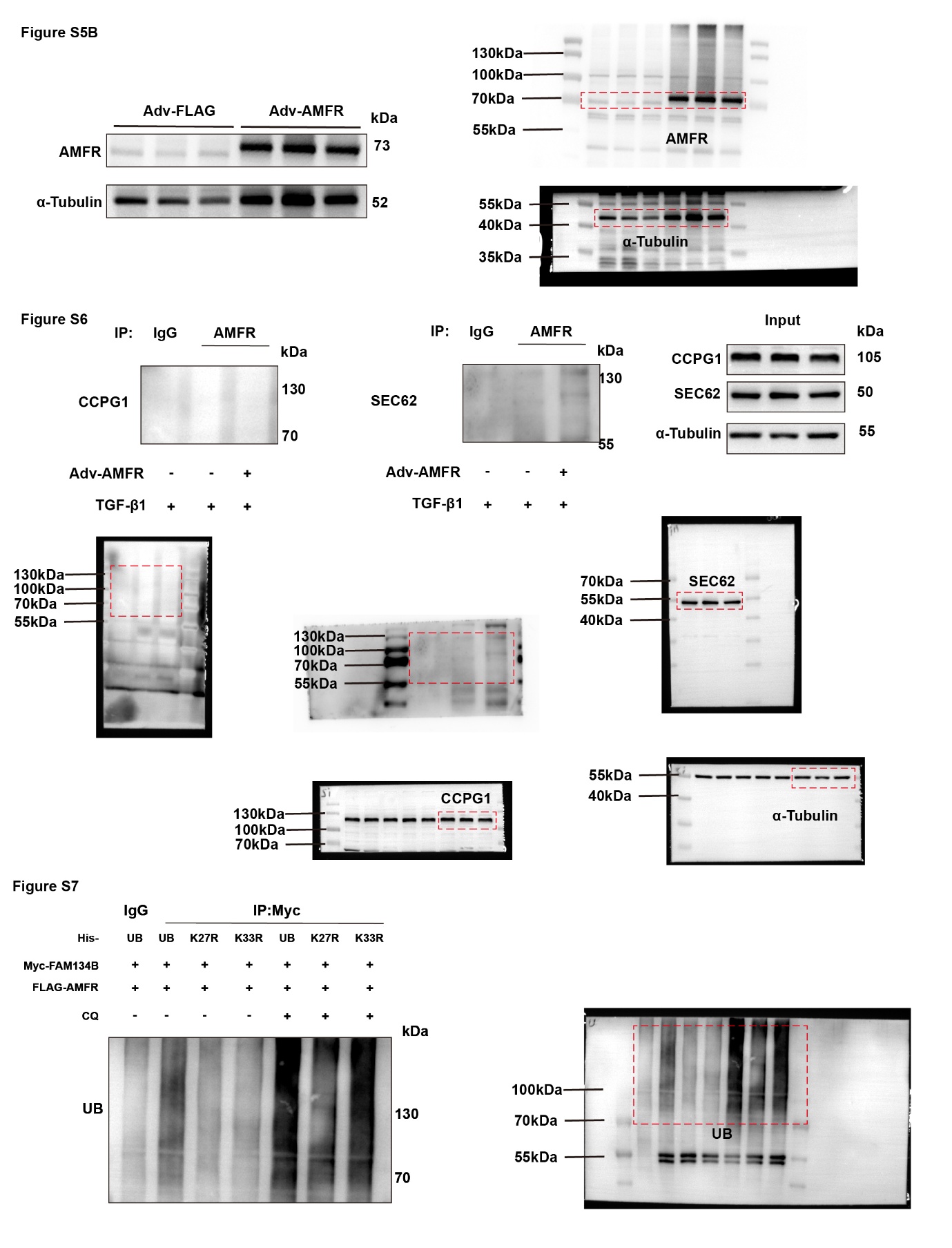


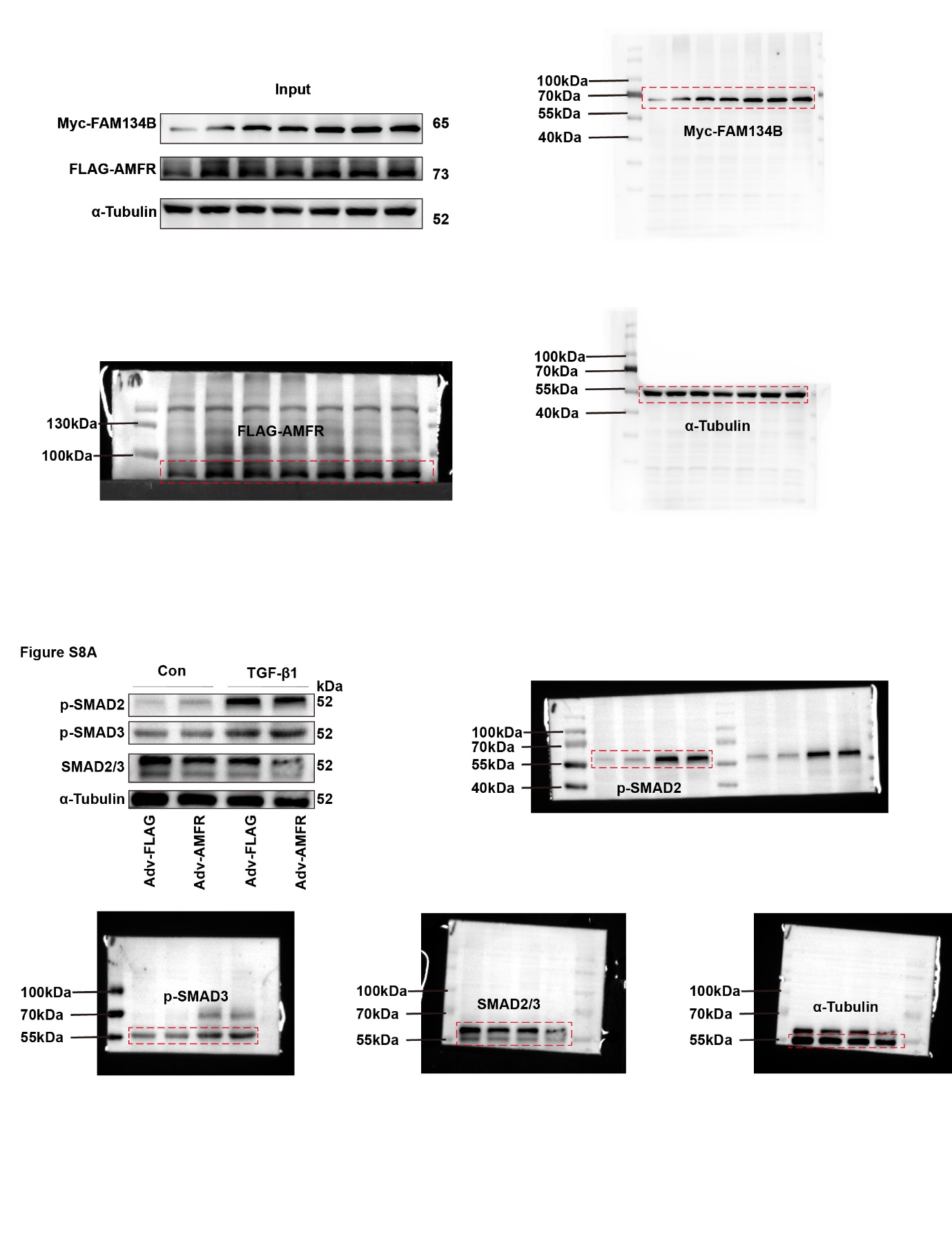


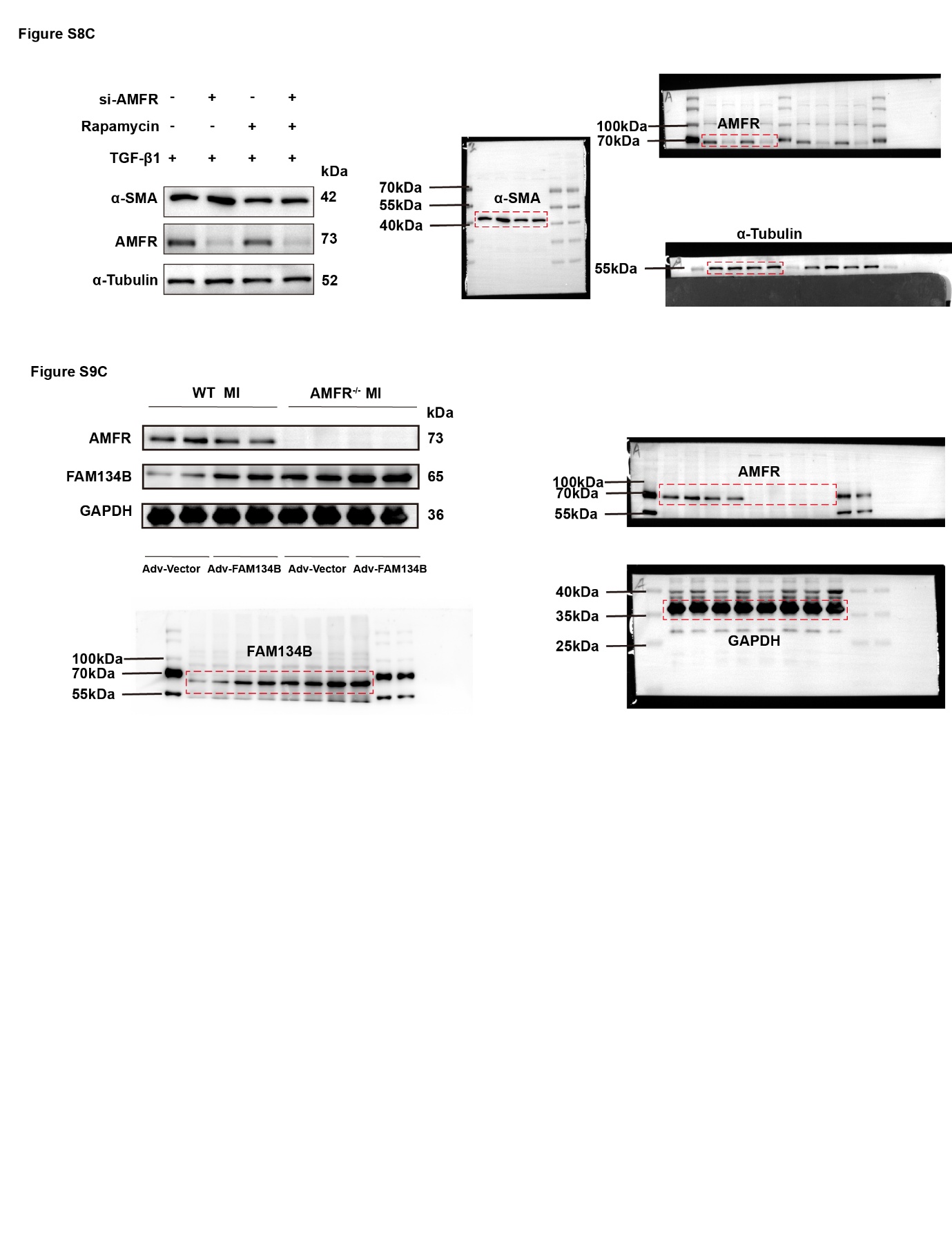

Supplement: Supplementary file 2 — Supporting Information [file ADVS-12-e04552-s002.docx]
